# Understanding Cyclists’ Visual Behavior Using Eye-Tracking Technology: A Systematic Review

**DOI:** 10.3390/s25010022

**Published:** 2024-12-24

**Authors:** Fatima Kchour, Salvatore Cafiso, Giuseppina Pappalardo

**Affiliations:** Department of Civil Engineering and Architecture, University of Catania, 64 Santa Sofia Street, 95123 Catania, Italy; giuseppina.pappalardo1@unict.it

**Keywords:** eye-tracking systems, cyclist safety, road safety, gaze behavior, visual attention, hazard perception, visual workload

## Abstract

Eye-tracking technologies are emerging in research aiming to understand the visual behavior of cyclists to improve their safety. These technologies gather real-time information to reveal what the cyclists look at and how they respond at a specific location and time. This systematic review investigates the use of eye-tracking systems to improve cyclist safety. An extensive search of the SCOPUS and WoS databases, following the PRISMA 2020 guidelines, found 610 studies published between 2010 and 2024. After filtering these studies according to predefined inclusion and exclusion criteria, 25 were selected for final review. The included studies were conducted in real traffic or virtual environments aiming to assess visual attention, workload, or hazard perception. Studies focusing on other types of road users or participants not involved in active cycling were excluded. Results reveal the important impact of road elements’ design, traffic density, and weather conditions on cyclists’ gaze patterns. Significant visual workload is imposed mainly by intersections. Along with the valuable insights into cyclist safety, potential biases related to small sample sizes and technological limitations were identified. Recommendations for future research are discussed to address these challenges through more diverse samples, advanced technologies, and a greater focus on peripheral vision.

## 1. Introduction

Cyclists represent one of the most vulnerable groups of road users. They are the only category of road users whose fatality rate has not decreased since 2010. Approximately 2000 bicycle-related fatalities and numerous serious injuries were reported to the authorities in Europe in 2022. Accordingly, bicycles account for one out of every ten recorded fatal crashes, and the majority of bike fatalities happen in crashes with motorized vehicles [1]. Additionally, there has been no reduction in the percentage of serious injuries recorded in incidents involving cyclists; rather, it climbed from 7% in 2010 to 9% in 2019 [2], and most of these injuries happen in single bicycle crashes [1]. However, because these crashes are likely not being reported regularly, the volume of this percentage could be significantly higher [3].

Crashes between cyclists and other vehicles usually happen when different road users interact, such as at crossings, junctions, and road segments with no designated cycling facilities [4]. Approximately two-thirds of cycling fatalities in the EU happen over long stretches of road, and nearly one-fifth happen at a roundabout or intersection. The percentage of bike deaths is lower on longer road segments and significantly higher at crossings when compared to all road fatalities [2]. The two types of crash scenarios that account for the majority of fatalities and serious injuries in bicycle crashes are known as crossing scenarios, in which the bicycle and the motor vehicle are traveling in perpendicular directions, and turning scenarios, in which the cyclists turn left (or right in the UK) in front of the motor vehicle [5].

On the other hand, the main contributing factors to single bicycle crashes are proportioned as follows: 60% are related to skidding on ice or water, 24.9% are related to road design (curbstones, obstacles near the road, road maintenance), and a smaller proportion are due to cyclists’ condition and behavior (alcohol, speed, distraction) [6].

To mitigate the risks, understanding the behavior and decision-making processes of cyclists is crucial for developing safer transportation infrastructure and implementing efficient safety measures. Because most of the information received while navigating the roads is visual, understanding where the cyclist looks and how their environment affects their behavior is essential to prevent crashes by taking suitable countermeasures. Eye-tracking technology has evolved into a useful tool for studying how cyclists perceive and interact with their surroundings because it records gaze data in real time. Researchers can learn more about the elements that affect cyclist safety, such as weather, traffic density, and road design, by examining visual behavior.

Despite the growing number of research studies on bicycle safety, comparatively little has been revealed about the application of eye-tracking technology in this area. Although existing studies have demonstrated an interest in understanding visual attention to enhance safety, a thorough synthesis of the data is still required to guide future investigations and urban planning strategies. This systematic review seeks to fill the gap by investigating the use of eye-tracking technologies to evaluate the visual behavior of cyclists. It aims to provide an overview of the current literature, highlight significant trends and limitations in the application of eye tracking for bicycle safety, and present recommendations for further studies.

## 2. Methodology

This section presents the methodological approach employed to conduct the systematic review, from setting the research question and extracting relevant articles from different databases to selecting studies included in the review.

### 2.1. Defining the Methodological Approach and Key Steps of the Review

This study intends to summarize the literature on eye tracker technology and identify the key challenges involved in their applications and the key opportunities these technologies will bring to the specific field of application for cyclist behavior and safety.

**Figure 1:**
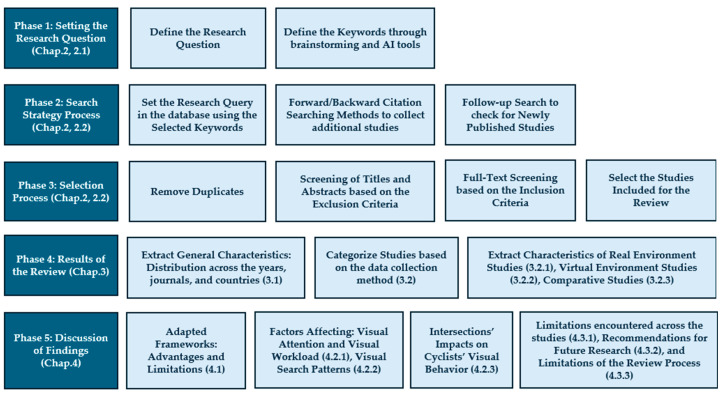
Key steps of the systematic review.

To achieve this aim, the study adopts a systematic literature review approach using the PRISMA (Preferred Reporting Items for Systematic Reviews and Meta-Analyses) [7] protocol to improve the study’s effectiveness and replicability. The flow chart in Figure 1 presents the phases of the systematic review, and it is also the formal structure of the paper.

### 2.2. Search Strategy and Selection Process

In November 2023, a narrow search was conducted in the two databases, SCOPUS and WoS (Web of Science), to investigate the use of eye-tracking technology for cyclist safety in the context of road safety. The SCOPUS and WoS databases have excellent coverage of appropriate disciplines and high-quality indexed publications. They afford good indexing, especially in peer-reviewed journals, and access to quality research articles across a wide range of topics associated with road safety and eye-tracking technology. It is important to note that SCOPUS and WoS also include content from IEEE and PubMed databases, thus ensuring the availability of high-quality and relevant studies.

First, the search strategy involved using a set of keywords obtained by brainstorming and asking advanced AI tools (ChatGPT-3.5) for keywords appropriate to the research question, focusing on how eye-tracking technology can help improve our understanding of cyclist behavior and road safety.

The search query set for the databases is as follows: (TITLE-ABS-KEY (“eye tracker*” OR “eye tracking*” OR “eye tracking system*” OR “eye tracking applications” OR “eye tracking glasses” OR “eye gaze data*” OR “real-time gaze data*” OR gaze* OR “measur* eye position” OR “measur* eye movement” OR “eye movement*” OR “visual attention” OR “gaze data*” OR “Driver’s visual attention” OR “Cyclist’s visual attention “) AND TITLE-ABS-KEY (safe* OR “road safety” OR “safe road*” OR “cycl* safety” OR “bicycl* safety” OR “safer road*” OR “safer road*” OR “bik* safety” OR “micro-mobility safety “ OR “ traffic safety”) AND TITLE-ABS-KEY (cycl* OR bike* OR bicycl* OR “cycl* user*” OR “bik* user*” OR “bicycl* user*” OR “vulnerable road users” OR “cycl* rider” OR “bik* rider” OR “bicycl* rider” OR “micro-mobility road user*” OR micromobility* OR “ cyclist behavior “)) AND PUBYEAR > 2009 AND PUBYEAR < 2024 AND (LIMIT-TO (LANGUAGE, “English”)).

After conducting the query on SCOPUS, we used Forward and Backward Citation Searching methods on the first eight relevant papers from SCOPUS, revealing a rich vein of literature. These eight papers were selected from the search query by applying the “sort by relevance” filter. In SCOPUS, Search Engine Relevance [8] is a complex statistical calculation that indicates how well the search terms and criteria match the text of the returned documents, helping to refine the search for better results. The query initially resulted in 221 documents. Using the Forward Citation Searching method, which involves exporting the list of papers citing the first eight relevant papers from SCOPUS, we found 24 documents. Conversely, the Backward Citation Searching method, which looks at the references cited in the first eight relevant papers and published between 2010 and 2023, yielded 202 documents that were selected. Then, from the WoS database, we obtained 142 documents. Additionally, a follow-up search in April 2024 found 14 in SCOPUS and 7 in WoS, extending the latest publication date to the first half of 2024. In total, we identified 610 studies.

The first step involved removing all duplicate studies, resulting in a total of 572 studies. Subsequently, the title and abstract of each study were screened and evaluated. Studies were then filtered based on the following exclusion criteria:

- It was a conference paper or thesis, because only peer-reviewed journal articles that underwent a thorough and rigorous review process were included, thus ensuring high reliability and validity of the selected research papers for the review.
- It was in the subject areas of medicine, neuroscience, biochemistry, pharmacology, etc. and it was not related to the general research question.
- It assessed behavior using any method other than eye-tracking.
- The eye-tracking study focused on cars and pedestrians but did not consider interactions with cyclists.
- It involved accidents/collisions related to car drivers.
- It focuses on road safety without mentioning cyclist safety.
- The screening of titles and abstracts was performed using the following two approaches:
- The title was assessed, and if it was clear that the report was in a different field than road safety (for example, medicine or neuroscience), the report was excluded.
- By giving ChatGPT-3.5 the title and abstract and asking the following yes/no questions: “Was eye tracker used in this study?”, “Were cyclists included in this study?”, and “Was cyclist safety assessed in this study?”.
- By evaluating the abstract thoroughly. Because the use of ChatGPT is experimental in systematic reviews, it is important to confirm the results of the AI tool.

This process resulted in 488 studies being excluded from the review and 84 studies being included in the full-text screening step.

The full-text screening process was executed for the included papers by setting the following inclusion criteria:

- It included interactions of cyclists with other road users (drivers of all types of motorized vehicles, pedestrians, motorists, and e-scooter riders).
- The eye tracker was used in the study to assess one of these outcomes, which are visual attention, visual workload, and hazard perception in the context of cyclist safety.
- The participants were actively cycling during the test, either in real traffic or using a bicycling simulator. Studies where participants were seated and only observing or reacting to recorded video footage or photographs on a computer screen were not included. Thus, the two included data collection methods were real traffic environments and virtual simulations, which will be discussed in detail in Section 3.

This process was conducted by employing the following two methods:

- Using the AI tool, ChatPDF, which allows researchers to upload their documents and then analyzes them and answers the questions asked by the reviewers. The yes/no questions were the following: “Does this study include interactions of cyclists with other road users?”, “Was the eye tracker used in the experiment to assess visual attention/ workload/ hazard perception in the context of cyclist safety?”, and “Are cyclists actively cycling during the experiment?”.
- When necessary, the reviewer read the text fully to answer the previous questions. ChatPDF provides answers with proof from the text by giving the page number and highlighting the evidence in the document. However, it may sometimes provide a doubtful response instead of a definitive yes or no. Thus, the answers of ChatPDF were checked carefully, and manual data extraction was performed when needed for accurate answers.

**Figure 2:**
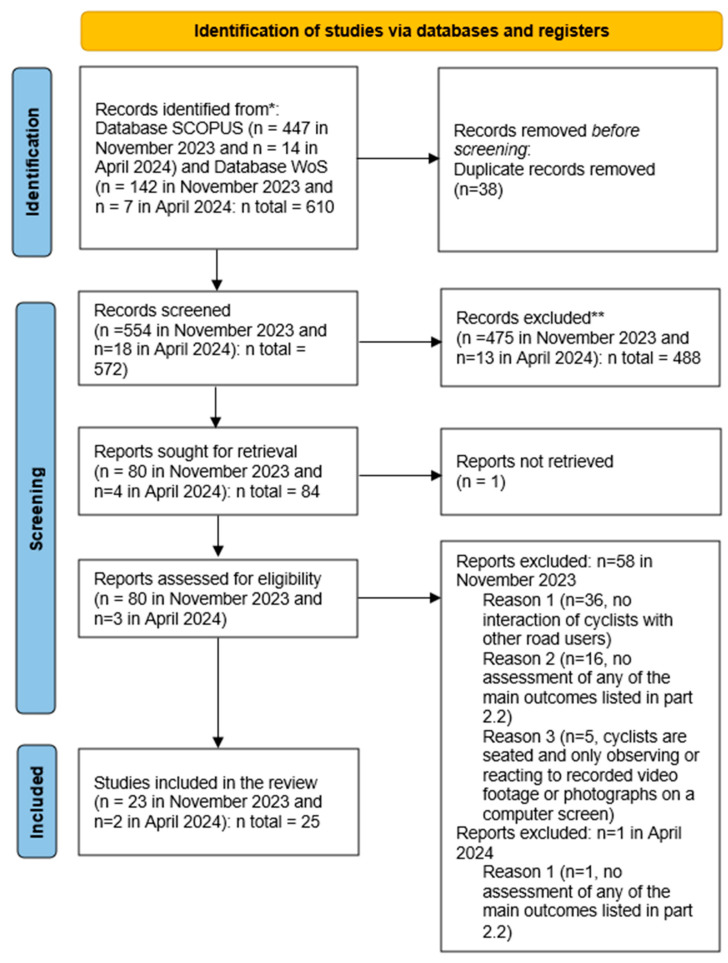
Study selection process based on the “PRISMA 2020 flow diagram for new systematic reviews”.

After screening, 25 studies were included in the review for further analysis and interpretation. A PRISMA flow diagram, shown in Figure 2, illustrates the selection process and provides a visual impression of the flow of information through different phases of the systematic review.

## 3. Results

This section is divided into two sub-sections: one presents the studies’ general characteristics, and the other discusses the results extracted from each study individually. Three summary tables with the main details related to all of the studies are available in Appendix A; Table A1 and Table A2 include the general characteristics and the outcomes interpreted, Table A3 and Table A4 contain the methodological information, and Table A3 presents the results of each study. They briefly describe the contribution of each study to understanding cyclist safety through eye-tracking.

### 3.1. Studies’ Characteristics

**Figure 3:**
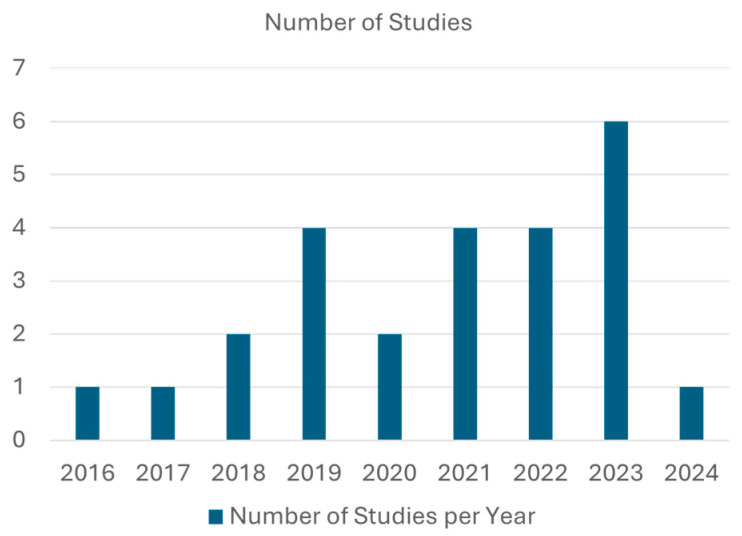
The distribution of studies across the years.

The 25 reviewed research studies differ in methodology, population, location, etc. These articles selected for the final review cover 8 years, from 2016 to 2024, with the highest number of studies, totaling six, in 2023. The distribution of these articles over the years is illustrated in the bar chart in Figure 3.

The review encompassed studies distributed across different journals, as shown in Table 1.

The majority of studies (*n* = 14) were conducted in Europe, whereas nine studies were conducted in North America and two in Asia. The demographic distribution is shown in Table 2.

### 3.2. Results of Individual Studies

This section describes the results obtained from the 25 studies, categorized based on their data collection method (real environment studies, virtual environment studies, and comparative studies including real and virtual traffic experiments). For each group of studies, the characteristics discussed are compiled as follows:

1. Samples’ characteristics.
2. Experimental design and procedure.
3. Data collection and analysis.

#### 3.2.1. Real Environment Studies

Eleven studies were conducted in a real traffic environment, where researchers gathered data as participants drove or interacted with real traffic. Thus, observations and measurements were conducted in real-world driving conditions.

1. Samples’ Characteristics

First, the average sample size across all the studies is 22, with a minimum size of 13 and a maximum of 50. The age range of the participants spans from 11 to 65 years, with an unweighted mean age of 29.55. Regarding gender distribution, five studies achieved gender equity among participants. Four studies had fewer female participants, with percentages ranging from 0% to 46.50%, while one study had more female participants, with a percentage of 52%. One study did not report the gender distribution of its sample. Most studies required participants to have normal or corrected-to-normal vision using contact lenses to ensure precise measurements, with two studies specifically mandating that participants not wear eyeglasses. Secondly, the sampling strategies employed across the studies included purposive, convenience, and mixed-method (purposive and convenience) sampling. The majority of studies (*n* = 9) utilized purposive sampling, where samples are selected based on the researcher’s discretion or specific criteria. Convenience sampling, where samples are chosen due to their easy accessibility or availability to the researcher, was used in one study. Additionally, a mixed-method approach combining purposive and convenience sampling, which involves selecting participants who are both easily accessible and meet the researcher’s criteria, was employed in one study.

Subsequently, participants were recruited through one or more of the following methods: from schools in proximity to the experiment’s location (*n* = 2), from the community of the city where the experiment was conducted (*n* = 2), by posting a questionnaire on social media and online platforms (*n* = 3), through university email lists, by using the university’s announcement board and website (*n* = 7), and by distributing flyers in the neighborhood surrounding the experiment’s location (*n* = 2).

Thirdly, participants were categorized as cyclists, drivers, or pedestrians, or they were required to experience the scenarios twice, once while cycling and once while driving or walking. Consequently, seven studies involved participants solely as cyclists, two studies involved only drivers, and one study included both cyclists and drivers. In studies involving cyclists, participants exhibited varying levels of cycling experience, ranging from cycling once a month for exercise and leisure to cycling daily. This diversity in cycling experience ensured the inclusion of both experienced and inexperienced cyclists, thereby enhancing the generalizability of the results. One study specifically focused on experienced cyclists. For studies involving only drivers, the sample was divided into two groups, cyclists, and non-cyclists, to assess the impact of cycling experience on driver behavior towards cyclists. The study that included both road user types required participants to engage in cycling and driving activities at least occasionally. Finally, one study required participants to participate as cyclists, electric scooter riders, and pedestrians, necessitating the completion of the test three times.

Detailed information on sample size, gender distribution, source of participants, road user type, and cycling experience is provided in Appendix A, Table A2, while information on sampling strategies is available in Table A3.

2. Experimental Design and Procedure

Firstly, study design approaches adopted in the literature were reviewed to find suitable methods that can be used to analyze eye-tracking data from road users in real traffic environments.

Eight studies utilized the observational/naturalistic methodology [9,10,11,12,13,14,15], which entails researchers observing and documenting behaviors, phenomena, or outcomes without manipulating any variable. This methodology was employed to examine the gaze behavior of participants (cyclists, drivers, pedestrians, electric scooter riders), focusing on their visual attention towards the areas of interest (AOI) [9,10,12,13,14,15]. Additionally, it was used to assess the visual workload experienced by cyclists [11], the visual scanning behavior of cyclists in relation to the spatial characteristics of the environment [11], and the visual scanning behavior of drivers when interacting with cyclists and other vulnerable road users (VRU) [12]. Study [12] also investigated driver gaze patterns while executing turns at urban intersections, while studies [13,14] analyzed cyclist gaze patterns during urban cycling. The relationship between cyclists’ visual behavior [10,11,12,13,14] and physiological measures [10,14], road conditions, participants’ characteristics, and environmental factors [11,12,13] was explored. Furthermore, studies [10,11] examined the relationship between gaze behavior and subjective risk perception.

In [16], Kircher K. et al. utilized a cross-sectional methodology to observe and collect data on the attentional demands and visual behavior of cyclists and drivers at a single point in time across three types of urban intersections: stop-controlled, unsignalized, and signalized.

In [17], Mantuano A. et al. implemented a quasi-experimental design to evaluate the impact of new signage on cyclists’ behavior and decision-making processes. Data were collected both before and after the signage implementation to analyze cyclists’ visual attention to signage and interactions with other road users. Unlike a traditional experimental design, participants were not randomly assigned but were selected based on their cycling experience and limited familiarity with the route to assess the influence of the new system on a group potentially representing “beginner cyclists”.

In [18], Kaya N. et al. employed a mixed-methods design, combining quantitative and qualitative approaches, to examine drivers’ gaze patterns and visual attention. The study analyzes coded video recordings to identify drivers’ scanning failures towards pedestrians and cyclists at urban intersections, providing both quantitative and qualitative insights.

In synthesis, the key aspects of visual behavior evaluation, addressed and analyzed in real traffic environments, are as follows:

- Visual attention towards different areas of interest (AOI).
- Visual workload in varied route environments.
- Visual scanning behavior and gaze patterns.
- Integration of kinematic, physiological, and subjective risk perception data with gaze data.

Characteristics related to the procedure and conditions of executing the different experiments are discussed. The researchers selected the driving scenarios based on the study’s objective. Studies [9,11,12,14,16,18,19] focused on various types of intersections to examine visual behavior at these critical locations. In contrast, studies [10,15,17] conducted experiments along complete urban routes with various characteristics to evaluate their influence on cycling and driving behavior in general and visual behavior specifically. The features examined included the following:

- Cycling path: on-street cycling lane with mixed traffic [10], on-street separate cycling lane visually distinguished by a colored pathway separating them from vehicles [10,13], on-street separate cycling lane separated from motorized traffic using a bollard [13], shared bicycle–bus lane [10], shared pedestrian–cyclist lane [10,11,14,17], designated bike lanes in urban traffic [12,18], narrow pedestrian/cyclist tunnel [11], shared pedestrian–cyclist–electric scooter rider lane in a park [15], and off-road cycling path [12,18], also known as a mountain biking trail or a dirt trail, designed and constructed for cyclists away from public roads and urban areas.
- Roadside parking areas: the presence of roadside parking areas along the cycling path [10,18].
- Intersections: Studies [16,18,19] included stop-controlled, unsignalized, and signalized intersections, while study [9] focused only on uncontrolled intersections. Studies [12,14] included only signalized intersections, whereas study [11] did not specify the types of intersections selected.

Selecting appropriate weather conditions is crucial in this type of experiment. Clear weather presents challenges due to variations in contrast, making it difficult to distinguish objects in areas of both bright light and shadow [19]. Additionally, direct sunlight interferes with the infrared signal of the eye tracker camera, causing frequent failures to capture pupil and corneal reflection (CR) positions [17]. Therefore, when using the Mobile Eye apparatus in outdoor settings, a cloudy sky is preferable to reduce signal loss and effectively preserve gaze information [17]. Studies [14,16,17] were conducted in cloudy weather during autumn and/or spring, while studies [9,12,13,15,18] reported dry and good conditions. Experiments in studies [13,19] were conducted in sunny and clear weather. One study aimed to assess safety and comfort while cycling in cold weather (temperatures between −11 °C and 9 °C) and snow-covered surfaces [10].

Detailed information about the study design, driving scenarios, and weather conditions is provided in Appendix A, Table A3.

3. Data Collection and Analysis

This section discusses the data metrics collected across the studies to analyze the cyclists’/drivers’ behavior in general, with a particular focus on visual behavior. Additionally, the equipment used for data collection and the software employed for data processing and analysis are detailed.

For each study, information about the data collected is provided in Appendix A, Table A3; the outcomes measured are in Table A4, and the results are in Table A5.

Three types of data were collected in the selected research, which are discussed as follows:

- Gaze data: collected using eye trackers. Table 3 presents the different gaze metrics measured and analyzed, along with their objectives.
- Kinematic data: obtained through GPS tracking, accelerometers, and gyroscopes, or by calculating the selected distance per time. Table 4 presents the different kinematic metrics collected and analyzed, along with their objectives.
- Subjective data: gathered through questionnaires, surveys, or interviews after the experiment. Similarly, Table 5 presents the subjective data collected and analyzed and their objectives.

All studies collected gaze data, while only three studies [10,15,16] collected kinematic data. Post-questionnaires and surveys were conducted in six studies [10,11,14,16,17,19].

Equipment used to collect gaze and kinematic data: Different types of equipment were used to collect gaze and kinematic data, which will be addressed in the following section.

For collecting gaze data, different types of eye trackers are reported: the “Pupil Pro” Eye tracker system (Pupil Labs GmbH, Berlin, Germany) with “Pupil” software (Pupil Capture and Pupil Player-Joint Release v0.3.8) [9], the “ASL Mobile Eye-XG” (ASL Eye Tracking, Billerica, MA, USA) Eye tracker system with “EyeVision” software [14,17], the “Pupil Labs Invisible” (Pupil Labs GmbH, Berlin, Germany) with a machine learning neural networks algorithm for traffic object identification developed by Epigram AS [19], “Tobii Pro-Glasses 2” (Tobii, Stockholm, Sweden) [10,13,15,16], the “SMI Eye Tracking Glasses V1” Eye tracker system (SensoMotoric Instruments, Teltow, Germany), in which gaze positions were manually marked on allocentric reference maps containing all stationary, traffic-related features [11], and the “Dikablis Eye Tracking Glasses 3” Eye Tracker (Ergoneers GmbH, Egling, Germany) with D-Lab software v3.50.8786.0 [12,18].

For collecting kinematic data, study [16] used two action cameras (Garmin Ltd., Olathe, KS, USA) equipped with GPS to collect data from cyclists, capturing the front view and the cyclist’s face, as well as the speed and position. For drivers, an instrumented vehicle, the “Volvo V60” (Volvo Car Corporation, Gothenburg, Sweden), equipped with the data logger “Video VBOX Pro” (RACELOGIC, Buckingham, UK), was used to collect the speed, position, and views from the forward, rearward, right-side, and driver’s perspectives. Study [10] collected the speed, power, and cadence using the “Garmin system” (Garmin Ltd., Olathe, KS, USA), which comprises various sensors. On the other hand, study [15] calculated the speed from recorded videos, using a simple formula: dividing the distance over time taken.

Regarding the duration of glances, it is important to note that certain studies have defined specific thresholds for saccades and fixations. Table 6 displays the thresholds specified in each study.

Subjective Data: Participants were primarily questioned about their test experience, focusing on their perception of risk and hazards, as well as any challenges that impacted their comfort and safety while driving.

Statistical Tests: The statistical tests employed across the studies can be categorized into three groups.

Descriptive statistical tests were used to describe data characteristics such as the mean, variance, and standard deviation. Studies [10,13,14,16,17] utilized descriptive statistics to calculate measures like the mean, the standard deviation, the frequency, and the percentage, and they also employed pie charts, histograms, and various graphical plots.

Model-based statistical tests were used to fit models to the data for predictions and conclusions. These included Generalized Linear Mixed Models (GLMMs) in studies [9,11,18], ANOVA tests in study [9], Wilcoxon Signed Ranks tests in studies [9,16], Z-tests in studies [11,16,17], logistic regression models in study [18], negative binomial models in study [12], and paired t-tests in studies [13,14].

Machine learning statistical tests involve algorithms for certain tasks, such as classification, segmentation, and tracking. Two algorithms were used in this review: the “Dwell-time fixation detection algorithm” in two studies [14,17], which is an unsupervised algorithm, with its tasks being segmentation and classification (it segments glances with a minimum duration of 100 ms, classifies them as fixations, and exports metrics like duration and inter-fixation duration), and The Neural Networks for Traffic Object Identification in the study [19], an algorithm that comprises two sub-algorithms. The FasteRCNN model consists of classifying objects (classification task) and identifying them within the video frame through segmentation, and the Kalman filter is used for tracking the objects over time. Thus, the neural networks algorithm can be used to automatically categorize the objects participants were viewing.

#### 3.2.2. Virtual Environment Studies

Eleven studies were conducted in a simulated environment. This type of experiment is designed to imitate real-world driving conditions. Participants engage with driving simulators that reproduce the sensation of operating a vehicle, incorporating visual, auditory, and tactile sensations.

Various types of virtual environment research were utilized, from low-level computer-based to high-fidelity and interactive virtual environments. The various types are explained below.

- Desktop Simulation: This involves simulation configurations whereby participants operate within an immersive environment, a non-immersive setup, physical interfaces for control, and without head-mounted displays or fully enclosed environments [20].
- Virtual Reality (VR) Cycling Simulator: a Virtual Reality (VR) Cycling Simulator, designed for hazard perception training in traffic and cluttered environments. It features real-time sensor integration and uses procedural content generation for dynamic scenario creation [21].
- High-Fidelity Virtual Reality Cycling Simulator: High-fidelity virtual reality cycling simulators join real-world-standard cycling equipment with that of virtual reality to bring about realistic riding conditions of cyclists for analysis of cyclist behavior in realistic surroundings [22].
- Advanced Immersive Bicycling Simulator: Utilization of high-fidelity visuals coupled with the situation in an extended manner is a technique that mimics closely the physical and visual dynamism of bicycling in the real world [23,24].
- High-Fidelity Driving Simulator: This type of simulator is characterized by its ability to provide a realistic driving experience that closely mimics actual vehicle behavior and environmental interactions [25,26,27,28].
- Advanced Immersive Driving Simulator: This is a combination of high-quality features that enable the provision of deep immersion and a responsive driving experience for effective studies of driver behavior in dynamic and emergency conditions [29].
- Full-Scale High-Fidelity Driving Simulator: A high-fidelity driving simulator that involves the use of realistic full-scale systems within the vehicle, advanced motion systems, and technologies involving visual and auditory immersion to create a highly precise and realistic driving experience [30].

**Figure 4:**
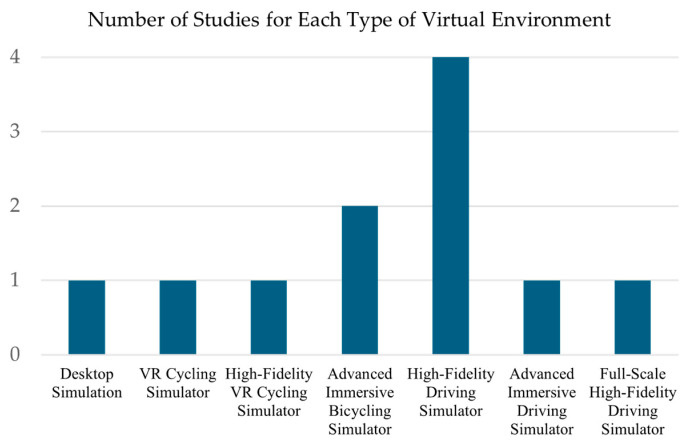
The number of studies for each type of virtual simulator.

Figure 4 shows the distribution of the studies across the different types of virtual simulators.

1. Samples’ Characteristics

First, the average sample size in all studies is 46, with a range from 5 to 130 participants. The age range of participants across all studies is between 10.83 and 65.8 years, with an unweighted mean age of 27.9. One study maintained gender equity. Four studies had fewer female participants, with percentages ranging from 0% to 46.50%, while four studies had more female participants, with percentages ranging from 57% to 70.77%. Among the eleven studies, four required participants to have normal or corrected-to-normal vision, while one study noted issues with eye tracking when wearing glasses. Two studies specified that only contact lenses were allowed, not eyeglasses. The remaining four studies did not provide information on vision requirements for participants. Secondly, the sampling strategies employed in the studies included purposive, convenience, and mixed methods (purposive and convenience). The majority of studies (*n* = 6) utilized convenience sampling. Four studies employed mixed-method sampling, while only one study used purposive sampling. Subsequently, participants were recruited through one or more of the following methods: from schools near the experiment’s location (*n* = 1), from the community of the city where the experiment took place (*n* = 4), through university email lists, announcements, and the website (*n* = 2), from the university campus (*n* = 2), and by distributing flyers in the neighborhood near the experiment’s location (*n* = 2).

Thirdly, participants were categorized as cyclists, drivers, or pedestrians, and some were asked to go through the scenarios twice. This resulted in four studies with participants exclusively as cyclists, six studies with participants exclusively as drivers, and one study that included cyclists and pedestrians. Among the studies involving cyclists, participants had varying levels of cycling experience, ranging from beginners to experienced cyclists. This diversity in participants enhances the generalizability of the results. In one study with drivers only, the sample was split into two groups, cyclists, and non-cyclists, to examine the influence of cycling experience on driver behavior towards cyclists. The other studies involving drivers required a minimum of one year of driving experience.

Detailed information on sample size, gender distribution, source of participants, road user type, and cycling experience is provided in Appendix A, Table A2, while information on sampling strategies is available in Table A3.

2. Experimental Design and Procedure

All studies were conducted in simulated virtual environments using an experimental design method. Researchers manipulated variables and observed their effects in controlled settings, selecting specific scenarios based on the aim of each study.

Springer-Teumer S. et al. [20] conducted a 2 × 2 mixed-design bicycle simulator, which included an experimental within-factor (system usage: WITH vs. WITHOUT Cyclist Warning System (CWS)) and a quasi-experimental between-factor (age group: younger vs. older cyclists) to evaluate the effects of the CWS on cyclists’ hazard perception through the analysis of gaze behavior.

In one study [23], Jashami H. et al. controlled environmental and roadway conditions to assess the visual attention of cyclists in commercial vehicle loading zones. Abadi M.G. et al. [24] created virtual scenarios to investigate the impact of pavement markings, truck traffic, and gender on cyclists’ visual attention.

Warner J. et al. [25] exposed drivers to different treatment configurations to assess the effects of engineering countermeasures on drivers’ visual attention during right-turn maneuvers at signalized intersections.

Kim A.J. et al. [26] manipulated variables (physiological arousal, attention to cyclists) within a controlled environment (a driving simulator) to observe their effects on driving behavior.

In the study [29], Zhao Y. et al. conducted driving simulator experiments to analyze driver responses to cyclists crossing from near and far sides.

Robbins C.J. et al. [30] involved the controlled manipulation of independent variables (such as road signs and vehicle types) in a simulated environment to observe their effects on dependent variables, such as drivers’ visual attention and behavior.

Fleskes K. et al. [27] manipulated independent variables (TOR proximity, relative bicycle position, secondary task engagement) to observe their effects on driver performance and interactions with cyclists.

In the study [28], Jannat M. et al. exposed participants to different scenarios in the simulated driving environment to assess driver behavior and visual attention.

Guo X. et al. [22] used immersive virtual environments to manipulate roadway design variables, analyzing their impact on the visual behavior and physiological responses of cyclists and pedestrians in controlled settings.

Zeuwts L.H.R.H. et al. [21] manipulated traffic scenarios within a VR simulator, examining young cyclists’ hazard detection and anticipation in a controlled setting that precisely measures their responses.

Characteristics related to the procedure and conditions of executing the different experiments are discussed. The driving scenarios simulated were tailored to the specific objectives of the study. Researchers controlled the environment by introducing critical events and conflicts among different road users. Some studies aimed to manipulate urban features to observe their impact on participants’ gaze behavior. The simulated driving scenarios included the following.

- Navigating Right-Turn Maneuvers at Intersections: used to explore the cyclists’ and drivers’ behavior in various intersection configurations [20,25,26,27,28].
- Cyclist–Vehicle Conflict Dynamics in Urban Environments: used to analyze the conflicts between cyclists and other vehicles (trucks, cars) in different urban settings (e.g., dooring scenarios, various loading zone configurations, lane configurations, intersection types) [20,21,23,24,25,26,29,30].
- Cyclist–Pedestrian Interactions: used to assess cyclists’ and pedestrians’ behavior at pedestrian crossings at signalized and unsignalized intersections [22]. Detailed information about the study design and driving scenarios is provided in Appendix A, Table A3.

3. Data Collection and Analysis

In this part, the data metrics collected used across the studies to analyze the cyclists/drivers’ behavior in general and their visual behavior in particular will be discussed. In addition, the equipment used to collect data and the software for data processing and analysis are also mentioned.

For each study, information about the data collected is provided in Appendix A, Table A3; outcomes measured are in Table A4, and the results are in Table A5.

Four types of data were collected in the selected research studies, which are discussed as follows:

- Gaze data collected using eye-tracking systems. These data are briefly summarized in Table 7.
- Kinematic data obtained through GPS tracking, accelerometers, and gyroscopes or by dividing the selected distance per time. These metrics are measured and analyzed, and their objectives are presented in Table 8.
- Subjective data, gathered through questionnaires, surveys, or interviews after the experiment. Table 9 shows the subjective data collected and analyzed, and their objectives.

Physiological data collected using sensors. Similarly, Table 10 presents the subjective data collected and analyzed and their objectives.

All studies collected gaze data, while only seven studies [20,21,22,26,27,29,30] collected kinematic data. Post-questionnaires and surveys were used in seven studies [20,21,22,23,26,29,30]. In addition, three studies [22,23,26] gathered physiological data.

Equipment used to collect gaze and kinematic data: The different types of equipment used to collect gaze and kinematic data will be addressed in the following section.

For collecting gaze data, the different eye-tracking systems used are the “SMI Eye Tracking Glasses 2” Eye tracker system (SensoMotoric Instruments, Teltow, Germany) with “SMI iView ETG” v2.7 software to record gaze data [20], the “ASL Mobile Eye-XG” eye tracker (ASL Eye Tracking, Billerica, MA, USA) [23,24,25,27,28] to collect gaze data, with a Logitech C920 HD Pro Camera (Logitech, Lausanne, Switzerland) to record videos for cyclists during the experiment [23], an eye-tracking camera “FOVIO” system (Seeing Machines, Canberra, Australia), used in a study [26] to collect drivers’ gaze data, a “Smart Eye Pro DX system” (Smart Eye, Gothenburg, Sweden), used to study the driver’s gaze movements in a study [29], and the “FaceLAB 5.0” eye-tracking system (Seeing Machines, Canberra, Australia), used to collect the driver’s gaze movements in a study [30]. One study [22] used a VR headset with eye tracking, “HTC VIVE Pro Eye” (HTC Corporation, Taoyuan, Taiwan), to collect cyclists’ and pedestrians’ gaze data, and the VR/AR eye-tracking glasses (Pupil Labs GmbH, Berlin, Germany) were used to collect gaze data in the study [21]. Seven studies [20,23,25,26,27,28,30] established the fixation threshold as 100 ms.

It is important to note that beyond standard gaze metrics, such as fixation and saccade durations or counts on areas of interest (AOI), more sophisticated metrics were also used. Study [21] introduced the “Entry time of the first fixation”, which measures the time between the onset of the event and the first fixation on the area of interest. In addition, two advanced metrics were employed uniquely in one study [22]: Stationery Gaze Entropy, which quantifies the level of focus of a person by measuring the gaze dispersion and concentration during a viewing period, and Gaze Transition Entropy, which measures the complexity of transitions between different gaze points. Meanwhile, the methods used for collecting kinematic data include measuring TTC (time to collision) through recorded videos as a metric for braking reaction behavior [20] and for collision avoidance behavior [27], measuring the driver’s travel time as a metric for approach behavior [30], and collecting driving measures, such as velocity, acceleration, and braking reaction time, from the simulator software [26,29]. In another study [21], the speed and braking rate were collected using the instrumented bicycle. A study [22] used the “SWEAR” app installed on a smartwatch to collect hand acceleration data, which were used to infer speed and steering data.

Subjective data: Participants were primarily questioned after the experiment to analyze their mental workload, perceived safety, comfort levels, and cycling/driving skills [20,21,23,26,29,30]. Additionally, participants were asked about their prior experience with different types of simulated experiments [21,23]. The majority of participants in study [23] reported previous experience with a bicycling simulator, whereas participants in study [21] had no experience with a virtual reality (VR) cycling simulator. Participants rated the virtual environment’s consistency with the real world [21,22,23]. The ratings indicated a high level of consistency between the real and simulated environments, demonstrating the simulator’s effectiveness and realism.

Physiological data: For collecting physiological data, the “Shimmer3 GSR +” sensor (Shimmer, Dublin, Ireland) was used to collect galvanic skin response (GSR) data [23] and electrodermal activity (EDA) [26] to understand the physiological state and the emotional arousal of cyclists [23,26], the “Zephyr BioHarness 3.0”(Zephyr Technology, Annapolis, MD, USA) was used to collect heart rate and breathing rate data in a study [26], the “SWEAR” app was used to collect heart rate data, and the VR headset was used to collect the horizontal head movement direction data [22].

**Figure 5:**
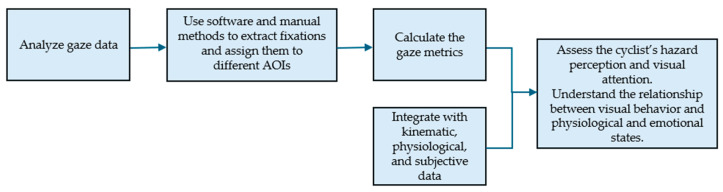
Steps of data analysis in simulated environment studies.

Figure 5 presents the diagram showing the steps of data analysis to evaluate visual behavior and its relationship with physiological and subjective data.

Statistical tests: The statistical tests in all of the studies are categorized into two groups. Descriptive statistical tests are used to describe the data characteristics, such as the mean, variance, and standard deviation. Studies [21,22,23,24,25,26,27,28,29] used descriptive statistics to calculate measures like the mean, the standard deviation, the frequency, and the percentage. They also utilized pie charts, histograms, and various graphical plots.

Model-based statistical tests were used to fit a model to the data to draw predictions and conclusions. These included ANOVA tests in studies [20,22,24,25,26,27,29,30], *t*-tests in studies [21,22,25,28,29], Correlation Analysis [21,22,26], Chi-square tests [21,28], linear mixed models (LMMs) [23], the F-test in study [29], the randomization test [26], and post hoc contrasts [26].

#### 3.2.3. Comparative Studies (Real Environment vs. Virtual Environment)

These studies involve conducting two experiments: one in a virtual environment and the other in real traffic. The main objective of these experiments is to compare the visual behavior of cyclists in both environments to evaluate the validity of virtual-based studies. One study [31] utilized a VR cycling simulator, whereas studies [32] and [33] employed an Advanced Immersive bicycling simulator.

1. Samples’ Characteristics

The average sample size is 21, ranging from 6 to 40 participants. In studies [31] and [33], the same participants cycled twice, once in real and once in simulated environments. Study [32] divided the sample in half, with one group cycling in real traffic and the other in a VR environment. The age range of participants in all studies is between 19 and 48.85 years, with an unweighted mean age of 30.44. One study [31] included only male participants, while the percentage of females was 45% in study [32] and 26% in study [33]. The sampling strategies employed in the studies consisted of purposive and convenience sampling. Studies [31] and [33] employed convenience sampling, while study [32] utilized purposive sampling. Participants were recruited through one or more of the following methods: volunteers from the community without specifying the source in [31] and [33], students from the university campus [32], and using social media platforms [32]. All participants were cyclists, with varying levels of experience, ranging from beginners to experienced cyclists.

See detailed information concerning the sample size, gender distribution, source of participants, road user type, and cycling experience in Appendix A, Table A2, and information about sampling strategies in Appendix A, Table A3.

2. Experimental Design and Procedure

All studies employed an experimental design method to compare gaze behavior in virtual traffic with real-world settings. Researchers manipulated simulator conditions to simulate real-world scenarios and then compared the findings with observations from field studies.

In the virtual scenarios, researchers in study [31] utilized machine learning algorithms to generate scenarios by training the model with input data and receiving feedback from the output. Studies [32,33] replicated the same circuit used in real cycling scenarios. The driving scenarios included the following.

- Cyclist–Vehicle Conflicts in Urban Settings: used to examine interactions in bus bays, near parking areas, and shared paths [31,32,33].
- Cyclist–Pedestrian Interactions: used to understand behavioral patterns at pedestrian crossings at intersections [31,33] and on shared paths [32,33].

Detailed information about the study design and driving scenarios is provided in Appendix A, Table A3.

3. Data Collection and Analysis

This section will discuss the data metrics utilized in the studies to analyze cyclists’ visual behavior. Additionally, we will mention the equipment employed for data collection and the software used for data processing and analysis.

For each study, information about the data collected is provided in Appendix A, Table A3; outcomes measured are in Table A4, and results are in Table A5.

The three research studies collected two types of data.

- Gaze data were obtained through eye trackers, and their characteristics and roles were examined in the data analysis and the results. These data are briefly summarized in Table 11.
- Subjective data were gathered via questionnaires administered after the experiment.

All studies included the collection of gaze data, with only one study [32] incorporating post-experiment questionnaires for data analysis.

Equipment used to collect gaze data: For collecting gaze data, various types of eye-tracking systems were used. Study [31] utilized the “FOVE VR Headset”(FOVE Inc., Minato-ku, Tokyo) in a VR experiment and the “SMI mobile eye gaze”(SensoMotoric Instruments, Teltow, Germany) in real traffic, “Pupil Core” (Pupil Labs GmbH, Berlin, Germany) was used in study [32] and the “Tobii ProGlasses 2” eye tracker (Tobii, Stockholm, Sweden), along with the “GoPro MAX” camera (GoPro Inc., San Mateo, CA, USA) to capture the view from the cyclists’ perspective used in study [33].

After collecting and synchronizing the data, the eye gaze metrics were analyzed using different methods based on the study’s methodology and objectives.

In study [31], researchers identified comparable scenarios of cyclists passing a bus in both VR and real-life settings. Hazard perception was assessed by analyzing fixations, saccades, and blinks from the first fixation until passing the bus using MATLAB R2020a v9.8. The agreement between VR and real-life gaze behaviors was evaluated using Bland–Altman plots.

In study [32], Pupil Core video data were analyzed frame by frame to identify areas of interest (AOI) and assign attention or inattention values for analyzing cyclists’ visual attention. These values were then compared between real and simulated experiments.

In study [33], researchers defined AOIs to categorize elements of the street environment and mapped gaze points onto a reference picture using Tobii Pro Lab. Gaze distribution was compared between real and simulated experiments using heat maps. Additionally, gaze metrics, such as the dwell time, visit count, average visit duration, and number of fixations per second on each AOI, were presented and compared between real and simulated experiments.

Subjective data: Study [32] collected data through two types of questionnaires: the “NASA Task Load Index” and a disease questionnaire to investigate the factors influencing cyclists’ safety and performance.

All studies used descriptive statistics tests to calculate measures like the mean, standard deviation, frequency, and percentage. They also utilized Bland–Attman plots, pie charts, histograms, and heat maps.

## 4. Discussion

This section elucidates the findings of Section 3 within two primary contexts. Initially, the frameworks employed across the studies are delineated. Subsequently, the principal outcomes of these studies, especially regarding visual attention and visual workload, are examined in detail.

### 4.1. Discussion of Adapted Frameworks

This review aims to elucidate cyclists’ visual behavior through the application of eye-tracking technology in various studies. It synthesizes the extant knowledge on how gaze data can be effectively utilized to enhance cyclists’ safety by evaluating their visual behavior. The review encompasses two primary data collection methodologies: real-world environments and virtual environments. This categorization provides diverse perspectives and assists researchers in selecting the most appropriate method based on their research objectives.

1. **Advantages and limitations of real and virtual environment studies.** Studies conducted in real traffic environments offer the advantage of yielding unbiased results, as participants are fully immersed in the actual traffic conditions. This contrasts with virtual environments, which impose controlled settings that may affect cyclists’ natural behavior [33]. However, ethical considerations constrain real traffic studies, particularly those involving distractions, hazards, and conflicts with other road users to assess their impact on cyclists’ visual behavior. This ethical issue can be mitigated in virtual and simulated studies [9,16,32]. The findings indicate that both real and virtual environments provide valuable insights into cyclists’ visual behavior, although there are notable differences in gaze distribution and fixation durations between laboratory and field studies [32].
2. **Importance of including different types of road users and different experience levels.** This review demonstrates that despite the focus on enhancing cyclists’ safety, the participant pool should not be restricted to cyclists alone [12,15,16,26,28,29,30]. Understanding the behavior of drivers and pedestrians towards cyclists is crucial, as interactions between drivers and cyclists predominantly occur at critical points, such as right-turn maneuvers, crossings, and on-road parking, while interactions between cyclists and pedestrians mainly occur at crossings and shared paths. Furthermore, including cyclists and drivers with varying levels of experience enhances the generalizability and applicability of the findings to broader populations [11,30].
3. **Main traffic scenarios included in the studies.** Regarding the driving scenarios employed, the findings indicate that the most prevalent scenarios across all study types were intersection-related and urban route driving scenarios. These results underscore the importance of examining cyclists’ behavior at intersections, which are considered the most complex and high-risk segment. These scenarios primarily assess the visual behavior, decision making, and interaction of cyclists and drivers with other road users at critical points [12,14,17,18,25,27,28]. Additionally, urban route scenarios included a mix of traffic conditions, roadway features, and interactions with pedestrians and other vehicles, providing comprehensive data on visual search strategies, visual search patterns, and risk management in urban settings [13,21,23,32,33].
4. **Relationship with subjective risk perception.** Various methods were employed across the studies to process and analyze gaze data. The general framework involved defining areas of interest (AOI), which are objects in the cyclists’ environment that may be observed during the experiment. Subsequently, gaze locations were extracted and overlaid on frames or the complete scene video. The frame-by-frame analysis [9], which involves extracting fixation data and manually associating each fixation with the relevant road factor, is the most consuming time method [14,17]. Overlaying fixation data on the scene video using software is the most effective method. Additionally, the introduction of machine learning algorithms is increasingly being adapted for extracting and processing gaze data. For instance, a classification task was performed using an unsupervised machine learning algorithm called the “Dwell-time fixation detection algorithm”, which classifies glances as fixations based on a determined glance duration threshold [14,17]. A pattern recognition algorithm was developed using neural networks to identify traffic objects that cyclists were looking at during the trip [19].
5. **Relationship with physiological measures.** The gaze data extracted and analyzed varied across the studies and were selected based on the specific aims of each study. The primary focus across all studies was on the fixation analysis, as it provides insights into various aspects of gaze behavior, such as visual attention, visual workload, visual search patterns, and strategies, as detailed in the previous sections. Generally, fixation data were used to assess how cyclists and drivers navigate through intersections and respond to signage and other road stimuli [13,15,17,24]. Additionally, this helps researchers evaluate the impact of different road layouts, vehicle types, and pedestrian activities on visual attention and explore how environmental conditions, like lighting or weather, affect the visual behavior of road users [9,12,16,28,32]. The principal gaze metrics calculated are discussed as follows:
  - Fixation durations and frequencies measure how long and how often participants fixate on specific points or objects, providing insights into what attracts the attention of drivers and cyclists and influences their behavior and decision making.
  - Fixation’s locations help researchers infer the priority of visual information and how environmental factors influence visual scanning behaviors.

### 4.2. Discussion of the Main Findings

This review highlighted the critical role of employing eye-tracking systems to evaluate the gaze behavior of various road users, including cyclists and those interacting with them. The included studies demonstrated the impact of several factors on each gaze behavior, such as visual attention and visual search patterns. This section is divided into three parts. The first two parts will examine the factors influencing visual attention, visual workload, and visual search patterns. The third one will discuss the role of intersection designs in shaping cyclists’ visual behavior and safety, given that intersections were the most frequently studied driving scenarios.

#### 4.2.1. Visual Attention and Visual Workload

Visual attention is the cognitive process of selectively concentrating on a specific aspect of the visual environment while ignoring other irrelevant information. Cyclists focus more intensely on a certain visual stimulus to process it rigorously and efficiently. Conversely, visual workload refers to the cognitive effort required to process visual information and perform visual tasks, representing the mental demands placed on an individual when interacting with visual stimuli.

The literature demonstrates the significant impact of environmental, infrastructural, and situational factors on the visual attention and workload of cyclists. These factors are discussed as follows.

**Environmental complexity:** It necessitates that cyclists balance their visual attention between central and peripheral fields of vision, particularly in complex environments, such as intersections or areas with high pedestrian density. Research indicates that an increase in pedestrian presence leads to greater environmental focus by cyclists [17]. Similarly, complex urban environments impose a high visual workload due to numerous exposed edges and potential hazards requiring continuous and rapid visual scanning. Intersection design also plays a crucial role; signalized intersections and those with poor signage impose greater cognitive loads by forcing cyclists to divide their attention among potential threats [11,14].

**Road surface and weather conditions:** The type and quality of the road surface significantly impacts visual attention. Adverse weather conditions and poor road surfaces add to the cognitive load by compelling cyclists to pay extra attention to avoid hazards [10].

**Signage and route guidance:** Proper signage improves navigation and safety by enabling cyclists to remain focused on the path, especially in complex traffic situations. New strategies in this direction have been identified that positively impact the visual attention of cyclists. Effective signage can reduce the visual workload by offering clear navigation cues, although significant cognitive challenges remain in complex traffic environments [19].

**Traffic and vehicle interactions:** The presence of other types of vehicles, especially large ones, like trucks, influences visual attention and workload. Cyclists tend to increase the frequency of their visual scanning in heavy traffic to ensure safety, leading to increased visual workload and short-duration fixations [24]. Visual attention and crash risks differ considerably with the relative positioning of cyclists to drivers, especially in right-hook scenarios. Approach angles and visibility influence driver response [28].

**Personal experience:** Experienced cyclists demonstrate more regulated visual scanning and longer fixation durations than inexperienced ones. They tend to bear a lower visual workload as they can manage their visual attention more expertly [11]. This indicates that experience is a significant factor in managing visual attention while cycling [11].

#### 4.2.2. Visual Search Patterns

Visual search patterns are affected by both the spatial complexity of the environment and the perceived risk. Areas with a high number of exposed edges necessitate more frequent visual scanning by cyclists, resulting in an increased visual workload and shorter fixation periods [11]. At intersections, cyclists display distinct visual patterns, with the number and duration of fixations varying according to intersection design.

#### 4.2.3. Intersections’ Impact on Cyclists’ Visual Behavior

Intersection design can significantly impact the visual behavior and safety of cyclists. The number and duration of visual fixations vary widely based on the intersection design. Signalized intersections and those with clear separation of cyclists from other traffic tend to reduce the cognitive load on the cyclists, facilitating more focused visual attention [14].

The complexity of intersection designs, particularly those with multiple lanes or obstructions, necessitates increased visual scanning by cyclists. Such areas demand a high visual workload and shorter fixation periods, which can negatively affect safety if not properly controlled [11].

Raised cycle tracks and other design features, such as physical separation from pedestrians or clear signage at intersections, have been shown to enhance visual attention and safety. Well-designed intersections help cyclists maintain the required T-shaped gaze pattern with frequent fixation points [13], reducing cognitive load and enhancing visual readiness for road situations. This predictability in gaze patterns contributes to safer navigation [13].

Furthermore, intersection design influences how drivers allocate their attention to cyclists. Protected intersection designs have been shown to reduce the potential severity of a crash by positively affecting driver behavior [25].

### 4.3. Limitations and Future Research

#### 4.3.1. Limitations

Some limitations have been widely noted and discussed in the studies. It is important to focus on them in future research to improve the quality of the results and increase accuracy. The limitations are classified and presented as follows.

1. **Sample size and power:** Small sample sizes were reported in many of the studies to have been one of the limitations, hence influencing the possibility to identify understated but potentially important effects [9,12,14,17,19,25].
2. **Generalizability:** Many studies are faced with the problem of their results not being generalizable to a broader population [10,11,19,29,32]. For instance, in study [14], there are challenges of their sample being diverse; hence, the applicability of its results will not work out for all age brackets and places. Similarly, Refs. [24] and [20] had findings whose usefulness demonstrated that the sample from simulating environments has limits in representing real-world situations.
3. **Selection bias:** Many of the researchers reported selection biases because the participants were drawn from particular demographics or institutions; see [10,12]. This limits the generalizability and applicability of the findings to a more general/different population.
4. **Limited use of machine learning in data processing and analysis:** The use of machine learning in data processing and analysis was limited and found only in three studies, where the neural networks algorithm was used to categorize the objects participants were viewing [19] and the “Dwell-time fixation detection algorithm” classified glances as fixations based on a determined glance duration threshold [14,17]. These tasks are among the first attempts to employ machine learning for working with gaze data.
5. **Eye-tracking technology limitations (information bias):** Several studies reported that eye-tracking technology lays a bias on the location of the fovea considering that foveal processing alone cannot support cycle control and safety due to limited information throughput [9,11,18]. Additionally, there exists a question regarding the applicability and reliability of eye tracking in dynamic cycling environments; see [13,18]. In addition, eye-tracking data may not accurately represent actual visual attention or conscious perception and therefore may introduce information bias. Thus, misinterpretation of eye-tracking data was noted as a risk in several studies, specifically by noting that the gaze may not imply visual attention or hazard perception [9,11,13,19].
6. **Confounding variables:** The most significant concerns were the external factors and the presence of the experimenters. For example, study [16] notes that the logging equipment and experimenters may cause potential confounding through alteration of participants’ natural behavior.
7. **Study design:** Fatigue and carryover effects were often noted in within-subject designs and thus affect data reliability, as in [23,28]. The lack of replication of the setup of peripheral vision was noted in studies, such as [17].

#### 4.3.2. Recommendations for Future Research

To address the previously mentioned limitations and enhance the frameworks and results of future studies, researchers from the reviewed studies proposed various recommendations for further implementation.

1. **Increasing sample size and diversity:** Numerous studies highlighted the need for larger and more diverse samples to improve the generalizability of results [9,12,14,16,17,19]. For example, study [17] recommends recruiting a broader range of individuals and conducting studies in different locations. Similarly, study [14] suggests increasing the sample size and including participants from varied backgrounds, ages, and genders. To analyze data using statistical tests and models, the average sample size for eye-tracking studies is 21 participants [34]. For studies implementing machine learning techniques, no previous studies have specified a certain number; however, because for each participant there is a large dataset, the sample size can be equal to or smaller than 21 (with a minimum of 10). Note that a reduction in the number of participants due to technical issues should be considered.
2. **Exploring peripheral vision and different urban settings:** Several studies emphasized the importance of considering peripheral vision and real-world conditions. This would allow researchers to gain a more comprehensive understanding of cyclists’ visual attention in various traffic conditions. For instance, study [18] suggests investigating the role of peripheral vision in VRU detection, while several recommendations highlight the need to conduct studies under varied urban layouts and traffic densities to understand how different environments impact cyclist behavior and safety [13,16,24,25].
3. **Behavioral studies under varied conditions:** Conducting experiments that examine cyclist behavior across different weather conditions, times of day, and traffic scenarios can provide more actionable insights into effective safety interventions [20,23,28]. As a proposed solution, long-term studies that capture data across different road types, weather conditions, and periods ensure an enhanced comprehension of cyclists’ adaptability to infrastructure and environmental changes. This type of study needs to define a clear objective and overall scope, select dependent and independent variables during long-term data collection, use eye-tracking data with advanced properties that overcome all weather conditions, and provide incentives for participants.
4. **Advancing data analysis through broader machine learning applications:** Study [19] suggests exploring machine learning to understand cyclists’ data demands better and enhancing existing data models by incorporating additional information. Moreover, machine learning can improve the accuracy of visual behavior models and help identify patterns more effectively. Because the road environment dataset contains significant variability, machine learning can also be applied to large datasets that include more factors from both the infrastructure and the cyclists’ environment [35,36]. As a result, a deeper understanding of cyclists’ behavior under various conditions can be achieved.
5. **Enhancing simulation fidelity:** Many studies recommend using advanced simulation technologies to better replicate real-world scenarios and reduce simulator-related biases [10,11,21,22,31]. For example, ref. [22] proposes new methods to improve the simulation experience by integrating development platforms like Unity or Unreal with the transportation simulation software (Synchro or Vissim) to simulate traffic networks, objects, and vehicle behaviors more credibly and create scenarios where different users can interact together, while [31] suggests adapting virtual reality settings to generate varying levels of traffic density.
6. **Validating and comparing results:** Conducting field tests to validate simulator findings and comparing gaze behavior across different environments were recurrent recommendations. Study [20] emphasizes the need for further simulator and field tests to validate the safety impact of the investigated systems. Additionally, ref. [11] suggests comparing gaze behavior and risk perception between different types of urban environments.
7. **Equipment and multimodal data collection:** Studies [9,18,19] recommend using more developed eye trackers with better storage and battery capacity and the ability to include peripheral vision. In study [19], the used eye tracker Pupil Invisible (manufactured by Pupil Labs) has a battery with up to 2.5 h of recording time and 40 h of recording storage, but as equipment has developed over the years, new eye-tracking glasses have up to 4 h of recording time and 25+ h of recording storage, such as Pupils Neon (manufactured also by Pupil Labs). Combining eye tracking with other methods, like EEG, emotion recognition, and interviews, can enhance the accuracy and depth of behavioral analysis [13]. In addition, ref. [22] highlights the importance of integrating heart rate data and behavioral responses with gaze data to understand the effect of stress levels and mental workload on the interactions of participants with the surrounding environment. Thus, such integrations can provide a holistic view of the cognitive and emotional states influencing cyclists’ gaze behaviors.
8. **Establishment of ethical standards:** Given the potential risks associated with real traffic research, establishing, and enforcing global ethical standards for road safety research is crucial [9]. This ensures the protection of participants while also maintaining the integrity of the research data.

#### 4.3.3. Limitations of the Review Process

The systematic review process imposes some limitations. First, the limited number of databases used and the restriction to selecting only peer-reviewed journal articles in English can introduce publication biases because some important research in other languages could be missed. Also, important findings from conference papers, theses, and other gray literature excluded could be missed. Second, the review included only studies between 2010 and April 2024; thus, some studies between April and September 2024 could be missed. In addition, some relevant papers may not be identified while conducting Forward and Backward Citation Searches. Finally, the use of AI tools in screening processes may result in selection bias. Because these tools are emerging in systematic reviews, several studies have evaluated their use, focusing mainly on ChatGPT and ChatPDF and highlighting their potential and limitations. ChatGPT helps in generating search strings for different databases and assists the researcher in screening titles and abstracts, thus enhancing and speeding up the first steps of the selection procedure [37]. However, it can sometimes fail to understand the meaning of keywords in search queries, miss Boolean operators, and degrade the quality of search results [37]. Suggestions from ChatGPT to decide whether to include or exclude a paper may be inconsistent or inaccurate, showing instances of erroneous content or missing important information [38]. Meanwhile, ChatPDF assists in full-text screening by providing semantic searches, evidence-based responses, and interaction with documents [38]. Its shortcomings stem from significant challenges in extracting and interpreting visual data, such as tables, figures, and graphs, and inadequate performance in extracting detailed methodological information related to data collection and sampling methods [39]. In addition, inaccurate answers were recorded when the title of the PDF document was missing [38]. To mitigate possible biases, researchers should use cross-checking and manual evaluation of AI-generated outputs [37,38], and maintain human judgment at critical review stages to guarantee the reliability and accuracy of results [39]. These practices help achieve a balance between ensuring rigorous and reliable systematic reviews and taking advantage of AI technologies.

## 5. Conclusions

This review emphasizes the importance of eye-tracking technology in studying cyclist behavior and improving cyclist safety. The examined studies provide various methods to analyze visual behavior using different gaze metrics and thus understand how cyclists interact with their environments, especially at critical locations, such as intersections, and in various urban environments under different traffic conditions. Analyzing the studies based on two data collection methods, real-world and virtual environments, shows important findings. The comparison of the two approaches highlighted the advantages and limitations of each one, mainly concerning the participants’ behavior, the ethical issues, and the possible biases in the data collected.

Interpreting the results enabled us to investigate the limitations across the studies and to suggest recommendations that help improve findings in further research. Issues, such as small sample sizes, selection bias, and limited applicability to broader populations, were identified. Furthermore, many studies encountered challenges, such as the limited information collected by eye trackers, which may not fully represent actual visual behavior, confounding variables that affect the naturality of the tests, and fatigue and carryover effects that influence participants’ behavior. In addition, the reliance on conventional data techniques, and the limited use of machine learning algorithms, restrict the ability to integrate additional factors into the road dataset and achieve more innovative results.

To address these limitations and improve the outcomes of future studies, several recommendations can be implemented. First, the generalizability of the results can be enhanced by increasing the sample size, including participants from diverse ages and backgrounds, and conducting tests in different locations that reflect varied infrastructure designs, weather conditions, and environmental conditions. Second, data reliability can be improved by exploring peripheral vision and its importance in visual behavior analysis. This can be achieved through the utilization of advanced eye-tracking technologies and the implementation of long-term studies that encompass a more comprehensive range of variables. Moreover, the creation of visual behavior models based on machine learning algorithms will facilitate advanced and accurate data analysis, as well as pattern identification. Integrating gaze data with physiological and subjective data provides a comprehensive understanding of cognitive demands and emotional and visual responses. Better simulation tools are also worth developing to create virtual environments that evoke a heightened sense of realism. When combined with real-world tests to confirm the results, these tools can help bridge the gap between controlled experiments and actual cycling conditions. Finally, adherence to ethical guidelines ensures participant safety and keeps the research trustworthy, paving the way for safer and better-designed cycling environments. 

## Figures and Tables

**Table 1:** The number of articles per journal.

Journal Name	Number of Articles
*Transportation Research Part F: Traffic Psychology and Behaviour*	4
*Case Studies on Transport Policy*	2
*Accident Analysis and Prevention*	2
*Journal of Safety Research*	2
*Traffic Safety Research*	1
*IEEE Access*	1
*Transportation Research Interdisciplinary Perspectives*	1
*Transportation Research Procedia*	1
*Journal of Architecture and Urbanism*	1
*Infrastructures*	1
*Sustainability*	1
*Cognitive Research: Principles and Implications*	1
*Sensors*	1
*International Journal of Automotive Engineering*	1
*Human Factors*	1
*Transportation Research Board*	1
*Journal of Advanced Transportation*	1
*Journal of Transportation Engineering, Part A: Systems*	1
*Virtual Reality*	1

**Table 2:** The distribution of studies across the countries.

Continent	Countries	Number of Articles
Europe	Belgium	1
	Germany	3
	Sweden Italy The Netherlands Finland Poland Norway UK	3 2 1 1 1 1 1
North America	USA	7
	Canada	2
Asia	Japan	1
	South Korea	1

**Table 3:** Gaze metrics’ characteristics and uses in data analysis in real environment studies.

Eye-Tracking Data	Characteristics	Objectives
Glance (fixation and saccades)	- Number - Duration - Percentage of glances to specific AOIs - Frequency categorized by direction and type of target	Investigate cyclists’ visual attention while listening to music and riding in urban areas, including, specifically, intersections [9], explore visual sampling strategies and their different purposes [16], understand the drivers’ behavior towards cyclists and road features when approaching intersections [12], understand how cyclists’ visual attention was allocated to different objects while cycling and interacting with other road users, and identify how visual scan patterns are affected by different urban features [13].
Fixation	- Duration - Number - Average point of gaze horizontal and vertical coordinates during fixation	Quantify visual workload during cycling [17], explore the relationship between visual attention and physiological responses (level of cortisol) [14], understand how spatial properties impact gaze patterns and risk perception [11], and understand the visual attention patterns [15]
Gaze	- Gaze direction accuracy - Pupil tracking accuracy - Visual angle accuracy	Analyze the driver’s visual attention allocation towards areas where potentially conflicting vulnerable road users (VRUs) could approach during driving maneuvers at urban intersections [18]
Eye-tracker videos Analysis		Explore cyclists’ behavior and understand how cyclists navigate challenging conditions and respond to environmental stimuli (when approaching a two-way cycling path when interacting with cars, on a U-turn, when faced with a red traffic light, etc.) [10].

**Table 4:** Kinematic metrics’ characteristics and uses in data analysis in real environment studies.

Kinematic Data	Objectives
Speed	Understand the effects of different road conditions (snowy and dry) on cyclists’ safety and comfort while cycling [10], examine the influence of speed on visual attention behavior and visual search patterns for pedestrians, cyclists, and e-scooter riders in shared road spaces [15], and explore how cyclists and drivers navigate intersections and the effect of speed on their ability to process visual information and respond to stimuli [16].
Power	Measures the amount of effort made by cyclists while pedaling to evaluate the physical demands on cyclists and their overall performance in different situations [10].
Cadence	Reflects a cyclist’s pedaling rhythm, revealing how they adapt their technique to maintain efficiency, control, stability, and safety in different conditions [10].
Position	Study how road users’ locations in intersections influence visibility, decision-making, and potential conflicts between cyclists and drivers [16].

**Table 5:** Subjective data characteristics and uses in data analysis in real environment studies.

Subjective Data	Objectives
Perceived safety and risks	Understand how cyclists interact in challenging conditions by analyzing their safety feelings while cycling in cold and snowy conditions [10] and understand the effects of the different levels of risk perception of cyclists on their behavior [11,14].
Comfort levels and difficulties encountered	Identify challenges faced in adverse weather conditions, thus improving cycling infrastructure [10], understand factors influencing decision making in different scenarios [16], understand how path quality affects cyclists’ comfort, and improve cycling facilities that are more challenging to navigate [17].
Perceived visual information	Identify the elements that capture cyclists’ attention at intersections [16] and urban routes [17], thus understanding the cyclists’ interaction behavior with other road users and their environment [16,17], and explore the effectiveness of implementing a new signage strategy and its influence on cyclists’ behavior [19].
Familiarity with test locations	Aims to understand the individual differences based on cyclists’ familiarity with the road and correlate them with cyclists’ visual behavior [11,14].
Cycling skills and habits	Classify participants and explore how different experience levels might influence their gaze patterns [11,14,16,17].
Effects of winter clothes	Examine how winter clothing affects cyclists’ visibility and mobility [10].

**Table 6:** The thresholds for fixation and saccade durations across the studies.

Study ID	Saccade Duration (Milliseconds)	Fixation Duration (Milliseconds)
[9,16]	<200	>200
[17]	(0–80)	(80–500)
[12]	<100	>100
[15]	<200	>200

**Table 7:** Gaze metrics’ characteristics and uses in data analysis in virtual environment studies.

Eye-Tracking Data	Characteristics	Objectives
Fixation	- Duration: mean duration, average total duration, total duration - Number on each AOI - The “Entry time of the first fixation”	Assess the hazard perception of cyclists in response to different AOIs within their field of view [20] and the visual attention performance of cyclists as they navigate commercial vehicle loading zones [23], understand the effects of pavement markings, truck maneuvering, and gender on cyclists’ visual attention during conflicts with truck traffic [24], assess the distribution of cyclists’ visual attention to different AOIs during the test [25], assess if visual attention is linked to physiological arousal and changes in driving behavior [26], evaluate the drivers’ visual attention at junctions [30], assess the visual attention of drivers of automated vehicles when they interacted with cyclists during a right-turn maneuver after receiving a take-over request (TOR) [27], measure drivers’ visual attention during right-turn maneuvers in the presence of adjacent cyclists [28], and evaluate young cyclists’ hazard perception and anticipation ability in different traffic situations [21].
Saccade	- Number	
Gaze	- Gaze angle - Gaze location - Horizontal gaze direction - Pupil diameter - Stationary Gaze Entropy—Gaze Transition Entropy	Integrated with velocity, acceleration, and braking reaction time to understand the effects of these parameters on car–cyclist collision occurrence [29] to explore visual scanning patterns, the level of visual workload, and stress levels (inferred from pupil diameter size) of participants [22].

**Table 8:** Kinematic metrics’ characteristics and uses in data analysis in virtual environment studies.

Kinematic Data	Objectives
Speed/velocity	Assess how quickly participants approach hazards to indicate their level of risk-taking behavior [21], understand their comfort levels and reactions to roadway designs or traffic conditions [22], evaluate interaction dynamics between vehicles and cyclists, including scenarios like right turns and intersection maneuvers [27], and assess collision risks and safe driving practices [26,29].
Braking reaction time (BRT)	Evaluate how quickly cyclists respond to critical events with and without collision warning systems (CWSs), thus assessing CWSs’ effectiveness in enhancing reaction times to hazards [20] and evaluate how much time drivers need to initiate braking after cyclists become visible [29].
Time to collision (TTC)	Evaluate CWSs’ influence on cyclists’ hazard awareness and reaction [20] and understand collision risks during interactions, such as right-turn scenarios or automated vehicle take-over requests [27,29].
Cadence	Evaluate cycling efficiency, control, and safety in simulated traffic scenarios [21]
Steering	Analyze participant maneuvering to avoid hazards, indicating decision-making and control abilities [22], and evaluate how drivers adjust steering in response to cyclists, reflecting their attentiveness and interaction dynamics [26].
Position data	Analyze the interaction dynamics between vehicles and cyclists, particularly in right-turn scenarios [27].
Distance measurement to oncoming vehicles at junctions	Assess how the distance affects the driver’s visual attention and decision making, particularly in terms of when and how safely they pull out at the junction [30].
Driver performance data	Evaluate how drivers adjust their lane position, steering wheel position, accelerator pedal position, and brake pedal position when interacting with cyclists to ensure safe navigation and collision avoidance and analyze time headway to upstream objects and the time to lane crossing to understand drivers’ situational awareness and ability to react appropriately to the presence of cyclists [30].

**Table 9:** Subjective data characteristics and uses in data analysis in virtual environment studies.

Subjective Data	Objectives
Perceived Safety	Assess cyclists’ feelings of safety when using collision warning systems (CWSs), focusing on their confidence and sense of security in hazardous situations [20], and evaluate drivers’ perceptions of safety when interacting with cyclists, particularly in challenging scenarios, to understand the emotional and cognitive influences on behavior [26,30].
Mental Workload	Analyze the cognitive demands put on cyclists using the CWS to determine whether the CWS has a positive or negative effect on mental workload [20].
Realism and Simulator Experience	Evaluate the realism and user experience of virtual environments to ensure they effectively replicate real-life conditions while minimizing simulator sickness symptoms [21,22,23].
Emotional Responses and Self-Perception	Capture drivers’ feelings of anxiety, nervousness, and self-perceived skills when interacting with cyclists to understand emotional and cognitive effects on behavior [26].
Individual Differences and Cycling/Driving Skills	Gather demographic data and driving or cycling experience to better understand participant behavior, focusing on differences in reactions to road scenarios [23,29,30].

**Table 10:** Physiological data characteristics and uses in data analysis in virtual environment studies.

Physiological Data	Objectives
Heart Rate (HR)	Track cardiovascular responses to traffic conditions, potential hazards, and environmental stimuli, offering insights into stress, excitement, or anxiety levels during interactions or simulated scenarios [22,26].
Breathing Rate	Evaluate variations in drivers’ breathing patterns as indicators of stress or anxiety when interacting with cyclists, thus assessing emotional arousal [26].
Electrodermal Activity (EDA)/Galvanic Skin Response (GSR)	Monitor skin conductance changes to evaluate physiological arousal or stress, helping to analyze participants’ emotional responses during challenging traffic scenarios [23,26].
Head Movements	Study participants’ head movement patterns to understand their visual scanning behavior and awareness of potential hazards [22].

**Table 11:** Gaze metrics’ characteristics and uses in data analysis in comparative studies.

Eye-Tracking Data	Characteristics	Objectives
Fixation	- Percentage of fixations - Duration: mean dwell time (mean fixation duration), total fixation duration - Fixation counts on each AOI	Assess the hazard perception skills of cyclists [31], analyze cyclists’ visual attention [32], and assess the difference in gaze distribution and fixation durations and numbers [33]. In all studies, results were compared between real and simulated experiments.
Saccade	- Percentage of saccades	
Blink	- Percentage of blinks	

## Data Availability

Not applicable.

## Appendix A

**Table A1:** General characteristics—part 1.

ID, Ref.	Objective(s) (Obj)	Research Question (RQ)/Hypothesis (Hyp)	Country	Sample Size
1, [9]	Examine the impact of listening to music on the visual behavior of teenage cyclists in real traffic environments.	Do cyclists counterbalance the reduced auditory input by amplifying their visual attention?	The Netherlands	14
2, [16]	Use the Minimum Required Attention (MiRA) theory and the Salience, Effort, Expectancy, Value (SEEV) model to examine whether attentional demands put on drivers and cyclists are different in urban intersections and how difficult it is to fulfill these requirements for the two road user groups.	It is assumed that drivers and cyclists experience differing levels of attentional demands within the same environment.	Sweden	23
3, [17]	Investigate cyclist gaze behavior in a real urban environment on bicycle tracks that are exclusive or shared with pedestrians.	This experiment enables analyzing quantitatively cyclists’ gaze patterns and understanding the factors that affect their trip.	Italy	13
4, [19]	Obj 1: Explore innovative approaches for evaluating the impact of route guidance measures on cyclists within actual traffic conditions. Obj 2: Assess the usability and traffic safety implications of the newly implemented wayfinding system.	RQ 1: How beneficial was the implementation of the new signage strategy by the Oslo City Council? RQ 2: To what extent did the Oslo city council’s adoption of the new signage strategy prove beneficial?	Norway	20–28
5, [10]	Examine cyclists’ behavior, their interactions with road users and, their response to various aspects of the road surface and geometric layout, using an instrumented bike in bad weather.	How do different road surface characteristics and geometric designs influence cyclists’ behavior, perception, and interactions with other road users in extreme weather conditions?	Sweden	22
6, [11]	Investigate the effects of subjective risk perception and spatial characteristics of a location on gaze behavior during urban cycling.	Hyp 1: Cyclists consider locations with increased spatial complexity and numerous obstacles that obstruct the line of sight as more unsafe, as they inhibit the early detection of potential hazards. Hyp 2: The spatial complexity of a location and the perceived level of risk do not necessarily dictate how long an object is fixated upon, but they significantly influence the ability and capacity to look ahead. In spatially complex environments, cyclists may need to divert their gaze in various directions and anticipate potential hazards from multiple angles.	Germany	20
7, [18]	Obj 1: Identify the prevalence of drivers’ visual scanning failures towards areas where conflicting vulnerable road users (VRUs) could approach at urban intersections. Obj 2: Assess how these scanning failures are moderated by cycling experience for non-novice drivers.	Hyp 1: Experienced drivers who regularly use a bicycle as a transportation tool will have fewer visual scanning failures towards areas where conflicting vulnerable road users (VRUs) can approach compared to drivers who rarely or never use a bicycle for transportation. Hyp 2: Drivers will exhibit more visual scanning failures at intersections with a history of crashes compared to those without a crash history.	Canada	26
8, [12]	Obj 1: Provide a more detailed quantification of the areas where drivers direct their attention, aiming to enhance comprehension of human factor challenges related to attention distribution at urban intersections. Obj 2: Examine potential discrepancies between drivers who are cyclists and those who are not.	How do drivers distribute their visual attention to vulnerable road users while navigating urban intersections?	Canada	26
9, [13]	Obj 1: Characterize the eye movement patterns of cyclists. Obj 2: Explore possible correlations between the eye gaze behavior of cyclists and urban environmental features, such as elevated cycle paths, physical barriers, land use, and pedestrian volume.	RQ: Study how urban features like elevated cycle paths, physical barriers, land usage, and pedestrian density impact the gaze patterns of cyclists. Hyp: Cyclists would exhibit specific gaze behaviors influenced by different urban characteristics.	Korea	50
10, [14]	Understand how design characteristics of intersections moderate how cyclists of various experience levels negotiate the intersections, as measured by the number and duration of fixations.	How do cyclists visually interact with the environment, and how do certain factors, such as experience level and intersection design, influence their visual attention and decision-making?	Italy	13
11, [15]	Investigate the visual attention and speeds of pedestrians, cyclists, and electric scooter (ES) riders when using shared roads.	Compare the visual attention and speeds of pedestrians, cyclists, and electric scooter riders in shared road environments to understand differences in behavior and potential hazards.	Poland	12
12, [20]	Assess the impact of the cyclist warning system (CWS) on behavioral indicators (braking reaction, gaze behavior) and subjective ratings of perceived safety and mental workload.	Investigate the effects of a CWS on a behavioral level and its users’ assessments, considering age-related effects.	Germany	51
13, [23]	Analyze the physiological response and visual attention of cyclists in commercial vehicle loading zones to understand how different environmental and roadway conditions affect cyclist stress levels.	Determine how the size of the commercial vehicle loading zone influences the stress responses of cyclists, considering factors like the presence and position of a courier and loading zone accessories.	Oregon, USA	48
14, [24]	Investigate the impact of pavement markings, truck traffic, and gender on the visual attention of cyclists during conflicts with truck traffic.	RQ 1: Do pavement markings and truck maneuvers affect the visual attention of cyclists? RQ 2: Does cyclist gender influence cyclists’ visual attention during conflicts with truck traffic?	Oregon, USA	48 (7 excluded due to technical problems)
15, [25]	Evaluate the effects of engineering treatments on motorist behavior during right-turn maneuvers at signalized intersections, specifically focusing on the latter portion of the green phase.	Hyp 1: “The engineering intervention does not impact the average duration of fixation for right-turning drivers on areas of interest (AOIs) within the driving environment.” Hyp 2: “The engineering intervention does not influence the time-to-collision (TTC) values of right-turning motorists during near-collision or collision events.” Hyp 3: “The engineering intervention does not impact the velocity of right-turning motorists during near collision or collision occurrences.”	Oregon, USA	28
16, [26]	Investigate the impact of cyclist-evoked arousal and attention to cyclists on driving behavior in the context of driver–cyclist interactions.	Stronger physiological arousal and greater attention to cyclists will be associated with safer driving behavior.	Texas, USA	104
17, [29]	Obj 1: Investigate driver responses in emergency car-to-cyclist collisions. Obj 2: Understand the factors influencing collision occurrences in scenarios with different time to collision (TTC) values for cyclists approaching from near and far sides.	How do drivers react when cyclists intrude into a car’s path at an intersection?	Japan	31
18, [30]	Examine the visual attention and behavior of two categories of drivers with varying levels of experience in pedal cycling (cyclists and non-cyclists) towards vulnerable road users at intersections within a driving simulator.	Investigating the behavior and visual attention of drivers with differing pedal cycling experiences towards vulnerable road users at junctions in a driving simulator.	UK	Study 1: 40 Study 2: 40
19, [27]	Investigate how the presence of cyclists affects driver performance during automated vehicle take-over requests (TORs) in a driving simulator.	How do human drivers of automated vehicles interact with cyclists during a right-turn maneuver after receiving a take-over request (TOR)?	Oregon, USA	46 (3 excluded due to technical problems)
20, [28]	Investigate the driver’s visual attention and the risk of a right-hook crash in a simulated virtual environment at signalized intersections when a cyclist is present and in different circumstances that might affect the driver’s visual attention.	How do the relative positions of adjacent cyclists influence a right-turning driver’s visual search and mean total fixation duration on areas of interest in the driving environment?	Oregon, USA	67 (16 excluded due to technical problems)
21, [22]	Propose a framework for evaluating cyclist and pedestrian behavioral changes by integrating human physiological sensing within immersive virtual environments (IVEs).	How are participants’ stress levels and cognitive load impacted by different roadway settings and real-time events in the IVE?	Virginia, USA	5
22, [21]	Evaluate hazard detection and anticipation of overt and covert traffic situations in young cyclists using an immersive virtual reality bicycle simulator.	Assess the effectiveness of a virtual reality cycling simulator in measuring hazard anticipation skills in children.	Belgium	130
23, [31]	Develop and evaluate a virtual environment to assess cycling hazard perception skills.	The development and evaluation of a Cycling and Hazard Perception virtual reality (VR) simulator (CHP-VR simulator) to assess and potentially train hazard perception skills in cyclists.	Finland	6
24, [32]	Obj 1: Analyze the visual behavior of cyclists in both simulated and real scenarios. Obj 2: Understand the factors that could affect their performance and safety.	RQ: Compare the visual behavior of cyclists in simulated and real scenarios to highlight objective differences in behavior. Hyp: There will be variations in visual attention and workload between the two scenarios.	Sweden	40 (20 for the on-site test and 20 for the simulated experiment)
25, [33]	Evaluate and compare the gaze behavior in the field with that in a cycling simulator setup to assess the realism of the simulator in reproducing traffic behavior.	Hyp 1: More control glances at the roadway in the field study are expected compared to the simulation study. Hyp 2: More active gaze behaviors in the field study are anticipated in terms of the number of fixations per time unit.	Germany	19 Simulation study: 15 Field study: 14

**Table A2:** General characteristics—part 2.

ID, Ref.	Source of Participants	Mean Age (sd)	Female, %	Road User Type	Cycling Experience	Equipment
1, [9]	From a secondary school near the SWOV Institute for Road Safety Research in The Hague, Netherlands.	17.1 (0.5)	50	Cyclist	On average about 6.5 h of cycling per week	A mobile eye-tracking headset at a rate of 30 Hz
2, [16]	A web-based questionnaire, distributed via Facebook.	39 (14)	52	Cyclist and driver	Cycled and drove at least sometimes	1. For cyclists only: two action cameras equipped with GPS 2. For drivers only: a data logger that records speed, position, and the forward, rearward, right-side, and driver views 3. For cyclists and drivers: a head-mounted eye tracker
3, [17]	From the community of the urban center of Bologna.	25 (7)	3	Cyclist	46% cycle every day and 3 inexperienced cyclists	A mobile eye-tracking system with glasses at a rate of 30 Hz
4, [19]	Parents of children of a school.	N/A	N/A	Cyclist	Have some, but not much, cycling experience	A mobile eye-tracking system with glasses at a rate of 30 Hz
5, [10]	1. Emails sent to university students and staff. 2. Posts on Stockholm’s social media groups.	Male: 37.9 (13.4) Female: 32.4 (14.1)	36	Cyclist	For males: 30.9 (15.9) years of average cycling experience For females: 26 (15.8) years	1. Instrumented bicycle: IMU, GPS, and other sensors 2. Mobile eye-tracker glasses at a rate of 50 or 100 Hz, and a microphone to record ambient sounds
6, [11]	Student recruitment via the announcement board of a research center.	26.6 (7.46)	50	Cyclist	Experienced cyclists: mean hours: 5.44 (1.04)	1. Mobile eye-tracking system glasses at a rate of 60 Hz 2. LiDAR scanning
7, [18]	1. University email lists, public. 2. Online platforms. 3. Flyers distributed in the vicinity of the university campus.	42 (4.5)	50	Driver	13 cyclists and 13 non-cyclists	1. The instrumented vehicle “2014 Toyota RAV4 vehicle”, equipped with two dashboard cameras—one facing the road and the other towards the driver’s seat 2. Head-mounted eye-tracking glasses at a rate of 60 Hz
8, [12]	1. University email lists, public. 2. Online platforms. 3. Flyers distributed in the vicinity of the university campus.	42 (4.5)	50	Driver	13 cyclists and 13 non-cyclists	1. The instrumented vehicle “2014 Toyota RAV4 vehicle” 2. Head-mounted eye-tracking glasses at a rate of 60 Hz
9, [13]	From the website of Seoul National University.	24	50	Cyclist	The majority cycled once a month or several times a year for exercise and leisure	1. Mobile eye-tracker glasses at a rate of 50 Hz
10, [14]	University students.	25	23	Cyclist	Divided nearly equally between experienced and inexperienced cyclists	A mobile eye-tracking system with glasses at a rate of 30 Hz
11, [15]	Students at Politechnika Krakowska.	For males: 22.7 (1.3) For females: 22.6 (3.3)	41	Pedestrian, cyclist, and electric scooter rider	Frequent users of both bicycles and electric scooters (ESs)	Mobile eye tracker at a rate of 25 Hz
12, [20]	1. From university email distributors. 2. Contacts in the private network.	For the younger group: 25.5 For the older group: 65.8	For the younger group: 63% For the older group: 29%	Cyclist	Experienced (regular)	1. Bicycle simulator with a cyclist warning system (CWS) developed by Fraunhofer IVI (at a rate of 10 Hz) 2. Eye-tracking glasses at a rate of 60 Hz
13, [23]	Not explicitly mentioned; the majority of participants were university students.	Male: 36.45 (15.57) Female: 29.84 (7.48)	0.5	Cyclist	More than half of the participants were experienced cyclists	1. Bicycling simulator 2. Galvanic skin response (GSR) sensor 3. Mobile eye tracker at a rate of 30 Hz 4. A camera to capture the participants’ runs
14, [24]	By posting and distributing flyers across the OSU campus and the city of Corvallis, Oregon.	Male: 29.44 (13.07) Female: 28.52 (11.93)	0.57	Cyclist	Not mentioned	1. Full-scale bicycling simulator 2. Mobile eye tracker at a rate of 30 Hz
15, [25]	From the local communities neighboring Corvallis, Oregon.	38	36	Driver	Not mentioned	1. High-fidelity driving simulator 2. Mobile eye tracker at a rate of 30 Hz
16, [26]	From a university campus.	26.7 (9.9)	0.63	Driver	Driving experience of at least 1.5 years	1. High-fidelity driving simulator 2. Physiological sensor 3. Eye-tracking camera at a rate of 60 Hz 4. GSR sensor
17, [29]	Not explicitly mentioned.	40.3 (17.2)	0.0645	Driver	Not explicitly mentioned; they have a driving license	1. Immersive driving simulator 2. Eye-tracking systems
18, [30]	Not explicitly mentioned; selected based on their frequency of pedal cycling usage.	Study 1: 24 (6.8) for pedal cyclists, and 22 (2.1) for non-pedal cyclists Study 2: 25 (7.3) for pedal cyclists, and 22 (7.3) for non-pedal cyclists	Study 1: 57.5% Study 2: 60%	Driver	* Half of the participants cycle frequently * Driving license dated between a minimum of 1 month and a maximum of 263 months * Annual mileage between 50 and 10,000 mile	1. High-fidelity driving simulator 2. Eye-tracking systems
19, [27]	1. Flyers distributed across Corvallis, Oregon. 2. Emails sent to various campus groups and mailing lists.	30.7 (15.11)	0.465	Driver	Driving experience of at least 1 year	1. Full-scale bicycling simulator 2. Mobile eye tracker at a rate of 30 Hz
20, [28]	From the area neighboring Corvallis, Oregon.	30.24	0.4117	Driver	Driving experience of at least 1 year	1. High-fidelity motion-based simulator 2. Head-mounted eye tracker at a rate of 30 Hz
21, [22]	From the local community: residents in Charlottesville.	31 (3.4)	Not mentioned	Cyclist and pedestrian	Experienced in cycling	1. VR headset with eye tracking 2. Smartwatch that has the “SWEAR” app installed 3. Stationary bike 4. Bicycle simulator 5. Treadmill 6. Cameras
22, [21]	From three schools that volunteered.	11.37 (0.54)	0.7077	Cyclist	Just be able to ride a bike; no additional criteria	1. Instrumented bicycle 2. VR headset 3. Eye-tracking glasses at a rate of 120 Hz
23, [31]	Volunteering, with no mention of the source.	33.7 (8.5)	0	Cyclist	Experienced: on average, 3.8 ± 2.9 h of cycling per week	1. CHP-VR simulator: stationary bicycle 2. Headset to develop the VR 3. Speed sensor 4. In real traffic environment: eye-tracking glasses
24, [32]	1. Social media networks. 2. Posters within the universities where the tests were conducted.	For the on-site test: 35.15 (13.7) For the simulated experiment: 27.47 (4.5)	45	Cyclist	For males: 30.9 (15.9) years of experience For females: 26 (15.8) years of experience * No previous experience with bicycle simulator	1. Mobile eye tracker 2. Bicycle simulator 3. The NASA Task Load Index (NASA-TLX) questionnaire
25, [33]	Not explicitly mentioned (were involved in a prior study examining visual search behaviors while cycling on road).	26.31 (7.31)	26	Cyclist	Varied between cycling frequently (many times) per week and less often than once per week	1. Head-mounted eye tracker at a rate of 60Hz 2. Scene camera 3. Simulator

**Table A3:** Methodological characteristics—part 1.

ID, Ref.	Driving Scenarios	Environment	Study Design	Sampling Strategy	Data Collection Method	Experiment Duration	Variables
1, [9]	Intersections	Daylight and dry weather	Observational (naturalistic)	Purposive sampling	Real traffic environment	Average: 13 min	1. Number of glances 2. Glance duration 3. Glance percentage to intersecting roads to the right
2, [16]	Intersections	In autumn: cloudy weather	Cross-sectional	Purposive sampling	Real traffic environment	Not mentioned	1. Attentional requirement 2. Visual behavior 3. Information sampling strategies (looking forward, left, down, etc.) 4. Complexity levels of intersections 5. Gaze behavior of cyclists and drivers
3, [17]	Intersections	In autumn and spring: cloudy weather	Observational (naturalistic)	Purposive sampling	Real traffic environment	Not mentioned	1. Cyclist gaze behavior 2. Visual elements in the urban environment 3. Cycling path characteristics 4. Participant demographics
4, [19]	Intersections	Clear weather (creates challenging conditions)	Quasi-experimental	Purposive sampling	Real traffic environment	About 20 min	1. Independent variables related to the signage strategy: type of signage, location of signs 2. Dependent variables: cyclists’ route choices, gaze behavior, and interaction with road users and signage
5, [10]	Urban route	Cold weather with snow-covered surfaces	Observational (naturalistic)	Convenience sampling	Real traffic environment	Not mentioned	1. Road surface characteristics 2. Geometric design of the infrastructure 3. Cycling speed, power, and cadence 4. Cyclists’ behavior, perception of safety and comfort, and participants’ demographic information
6, [11]	Intersections	Good weather conditions	Observational (naturalistic)	Mixed method: convenience and purposive sampling	Real traffic environment	About one hour	1. Subjective risk perception 2. Vista space properties (openness, drift, and mean radial length) 3. Individual factors (such as cycling experience) 4. Gaze behavior parameters (fixation duration, sight vector lengths, gaze angles)
7, [18]	Urban route, intersections	Good weather conditions between April and May	Mixed-methods design: quantitative–qualitative	Purposive sampling	Real traffic environment	Average: 14 min	1. Cycling experience (cyclist drivers vs. non-cyclist drivers) 2. Demographic variables (gender, age, years of full licensure) 3. Driving frequency 4. Presence of family members who ride bicycles frequently 5. Eye gaze metrics: gaze direction accuracy, pupil tracking accuracy, and visual angle accuracy
8, [12]	Intersections	Good weather conditions between April and May	Observational (naturalistic)	Purposive sampling	Real traffic environment	Not mentioned	1. Driver Characteristics (such as cycling experience) 2. Signal status (green or red) 3. Areas of relevance for vulnerable road users 4. Driver gaze behaviors
9, [13]	Urban route	Sunny days between August and September	Observational (naturalistic)	Purposive sampling	Real traffic environment	About 50 min	1. Urban characteristics: - Raised cycle track - Physical separation - Land use - Number of pedestrians 2. Cyclists’ gaze behavior - Gaze allocation - Gaze dispersion
10, [14]	Intersections	Cloudy weather	Observational (naturalistic)	Purposive sampling	Real traffic environment	Not mentioned	1. Cyclists’ experience level 2. Eye gaze behavior: - Fixation duration - Number of fixations - First fixation distance 3. Intersection design characteristics 4. Physiological responses, such as cortisol activity
11, [15]	Urban route	Dry weather (late summer, early autumn)	Observational (naturalistic)	Purposive sampling	Real traffic environment	About 2h	1. Mode of transport (pedestrians, cyclists, electric scooter riders) 2. Fixation zones (horizon, sides, roadway, pedestrian, transport vehicle) 3. Fixation distances 4. Speeds 5. Traffic rule violations
12, [20]	Cyclist–driver interaction scenarios at intersections	Not available	Experimental	Convenience sampling	Virtual traffic environment using a bicycle simulator	Not mentioned	1. System usage (WITH vs. WITHOUT CWS) 2. Age group (younger vs. older cyclists)
13, [23]	Cyclist–driver interaction scenarios at vehicle loading zones	Not available	Experimental	Mixed method: convenience and purposive sampling	Virtual traffic environment using a bicycle simulator	Average: 15 min	1. Loading zone size 2. Courier position 3. Presence of accessories 4. Participant demographics 5. Number of fixations 6. Fixation duration
14, [24]	Cyclist–truck driver interaction scenarios	Not available	Experimental	Convenience sampling	Virtual traffic environment using a bicycle simulator	Not mentioned	1. Colored pavement markings 2. Truck maneuvering 3. Cyclists’ gender 3. Total fixation duration on pavement markings (TFD-PM) 4. Total fixation duration on the truck (TFD-Truck)
15, [25]	Cyclist–driver interaction scenarios at intersections with different engineering treatments (markings, signs, curb radius)	Not available	Experimental	Convenience sampling	Virtual traffic environment using a driving simulator	Not mentioned	1. Engineering treatments: - Regulatory signage - Intersection pavement marking - Smaller curb radius - Protected intersection design 2. Driver performance measures: - Fixation duration on areas of interest (AOI) - Time to collision (TTC) values at the time of near-collisions or collisions - Motorist’s velocity at the time of near-collision or collision
16, [26]	Cyclist–driver interaction scenarios	Not available	Experimental	Convenience sampling	Virtual traffic environment using a driving simulator	One hour	1. Driver performance data: - Steering wheel position - Accelerator pedal position - Brake pedal position - Velocity - Time to lane crossing - Time headway to an upstream object - Lane position 2. Physiological data (at baseline and when visually encountering a cyclist): - Heart rate - Breathing rate - EDA (electrodermal activity) 3. Eye movement data: - Saccades - Fixations durations 4. Self-reported measures from questionnaires
17, [29]	Cyclist–driver interaction scenarios	Not available	Experimental	Convenience sampling	Virtual traffic environment using a driving simulator	Not mentioned	1. Car and cyclist velocities 2. Distances from the car and cyclist to the point of intersection 3. Time to collision (TTC) 4. Braking reaction time (BRT) 5. Time when the cyclist is visible 6. Gaze behavior
18, [30]	Cyclist–driver interaction scenarios at T-junction	Not available	Mixed-methods design: - Experimental - Observational	Purposive sampling	Virtual traffic environment using a driving simulator	Not mentioned	1. Road Sign: “Give Way” and “Stop” 2. Group: pedal cyclists vs. non-pedal cyclists, 3. Drivers’ license 4. Annual mileage 5. Age range 6. Vehicle type: pedal cycle, motorcycle, and car 7. Distance to oncoming vehicles at junctions (near, medium, and far) 8. Proportion of fixations 9. Mean fixation duration
19, [27]	Right-turn maneuvers scenarios	Not available	Experimental	Mixed method: convenience and purposive sampling	Virtual traffic environment using a driving simulator	30 min	1. Relative bicycle position 2. Secondary task engagement 3. Driver’s speed 4. Track length 5. Driver performance measures: - Time to collision - The driver’s yielding behavior - Fixation data
20, [28]	Right-turn maneuvers scenarios	Not available	Experimental	Mixed method: convenience and purposive sampling	Virtual traffic environment using a driving simulator	About 50 min	1. The relative positions and speeds of adjacent cyclists 2. The presence of oncoming left-turning vehicular traffic 3. The presence of conflicting pedestrians 4. Eye movement data: - Number of fixations - Total fixation durations - Average fixation durations - Time of the first fixation within each AOI
21, [22]	Scenarios in urban route	Not available	Experimental	Convenience sampling	Virtual traffic environment (VR)	Not mentioned	1. Heart rate 2. Hand acceleration 3. Fixations 4. Horizontal gaze direction 5. Gaze Entropy: Stationary Gaze Entropy (SGE) and Gaze Transition Entropy (GTE) 6. Pupil diameter 7. Horizontal head movement direction
22, [21]	Hazards scenarios for young cyclists	Not available	Experimental	Mixed method: convenience and purposive sampling	Virtual traffic environment (VR)	Not mentioned	1. Gaze behavior: - Fixation rate (whether the participant fixated on the area of interest during the event) - Entry time of the first fixation (time between the onset of the event and the first fixation on the area of interest) - Dwell time on the area of interest (duration of fixation on the area of interest) 2. Cycling behavior: - Speed (mean speed of the participant during the event) - Braking rate (binary code indicating whether the cyclist braked for the presented danger within the timeframe) 3. Hazard detection 4. Hazard anticipation 5. Simulator sickness 6. Realism perception
23, [31]	Urban route	Dry weather conditions	Experimental	Convenience sampling (opportunistic)	Virtual traffic environment (virtual reality (VR))	Not mentioned	1. Gaze location: - Fixation - Blinks - Saccades 2. Participant characteristics (such as cycling experience) 3. Reaction times to hazards
24, [32]	Urban route	Dry weather conditions	Experimental	Purposive sampling	Comparative	Not mentioned	1. Visual attention 2. Workload 3. Fixation duration 4. Attention distribution 5. Cycling experience 6. Familiarity with the route 7. Subjective perception
25, [33]	Urban route	Dry weather conditions	Experimental	Convenience sampling	Comparative	Not mentioned	1. Gaze behavior metrics: - Number of fixations - Fixation duration - Gaze frequency - Gaze density - Fixation counts on areas of interest (AOI) 2. Areas of interest (AOI) 3. Eye movement patterns

**Table A4:** Methodological characteristics—part 2.

ID, Ref.	Outcomes Measured	Study ID	Outcomes Measured
1, [9]	Visual attention	14, [24]	The effects of pavement markings, truck maneuvering, and gender on cyclists’ visual attention during conflicts with truck traffic
2, [16]	1. Differences in attentional requirements between drivers and cyclists 2. Fulfillment of attentional requirements (if the demand was high or low) 3. Fulfillment of information sampling strategies (effort and timing needed) 4. Difficulty of fulfilling these requirements for both road user groups 5. Glance frequency per direction and target type	15, [25]	Changes in driver performance during right-turn maneuvers at signalized intersections: 1. Visual attention of motorists 2. Crash avoidance behavior 3. Potential crash severity
3, [17]	1. Proportion of fixation 2. Number and duration of fixations 3. Relative frequency of fixation on different elements 4. Cyclists’ visual workload	16, [26]	1. Time to fixate on the cyclist on the first overtake interaction 2. Peak response and differences in values of physiological data when encountering a cyclist 3. Relationship between the self-reported responses, physiological and eye-tracking measures, and simulator behaviors using a correlation matrix: attentional bias to cyclists, arousal due to encountering a cyclist
4, [19]	1. Impact of the signage strategy on cyclists’ route choices 2. Glance duration at road items 3. Attention to road users and signage 4. Overall navigational experience in urban environments	17, [29]	1. Number of collisions and avoidances 2. Behavior changes to avoid collisions 3. Compare the values of speed, BRT, and TTC between the collision group and the collision avoided group 4. Gaze location 5. Braking and swerving responses of participants 6. Notice time 7. Gaze angle
5, [10]	1. Perception of safety and comfort 2. Power and cadence during cycling 3. Visual attention using the eye tracker 4. Subjective evaluations of the cycling experience	18, [30]	1. Visual attention measures of drivers towards vulnerable road users at junctions 2. Differences in visual search behavior based on pedal cycling experience 3. Comparison of drivers’ behavior when pulling out or waiting at junctions
6, [11]	1. Traveling speed 2. Subjective risk perception 3. Fixation duration 4. Sight vector lengths 5. Gaze angles	19, [27]	1. Collision avoidance behavior: - Driver’s yielding behavior - Time to collision (TTC) with a cyclist 2. Visual attention: - Driver’s total fixation duration (TFD) on the cyclist - Driver’s first fixation on the cyclist in the roadway
7, [18]	1. Gaze locations: to identify patterns of visual attention allocation and potential scanning failures towards VRUs 2. Number of turns, categorized as instances of visual scanning failure across each combination of cycling experience and crash history 3. Number of turns, identified as instances of visual scanning failure across various types of turns	20, [28]	1. Driver’s visual attention 2. Risk of a right-hook crash
8, [12]	1. Driver glance patterns (temporal glance distributions, percentage of time looking at different AOIs, percentage of time looking by cycling experience) 2. Attention allocation to pedestrians and cyclists 3. Potential risks posed to vulnerable road users (glance allocation to visual scanning failure related to AOIs)	21, [22]	1. Changes in heart rate 2. Gaze behavior 3. Visual scanning patterns 4. Stress levels of participants in response to different roadway settings and events in the IVE
9, [13]	1. Distribution of cyclists’ gaze fixations 2. Fixation duration percentage based on different road factors 3. Impact of urban characteristics on cyclists’ gaze patterns	22, [21]	1. Performance in hazard detection tasks 2. Performance in hazard anticipation tasks 3. Simulator sickness scores 4. Realism perception scores 5. Gaze behavior metrics
10, [14]	1. Patterns of gaze behavior 2. Differences in visual attention between experienced and inexperienced cyclists 3. Impact of intersection design on cyclists’ visual behavior and decision making	23, [31]	1. Gaze behavior 2. Perception–action coupling in VR and naturalistic environments 3. The comparability of visual search behavior in VR and real-world cycling scenarios
11, [15]	1. Number of fixations 2. Fixation durations 3. Distribution of Fixations in different zones 4. Speeds of participants 5. Interactions with other road users 6. Traffic rule violations	24, [32]	1. Variations in visual attention between simulated and real scenarios 2. Differences in attention distribution 3. Fixation duration 3. Subjective perception of mental and physical workload 4. Level of frustration 5. Cognitive and physical workload
12, [20]	1. Braking behavior: braking reaction time 2. Gaze behavior - Gaze reaction time - Fixation duration - Fixation number 3. Mental workload 4. Perceived safety	25, [33]	1. Differences in fixations and visits between the field and simulation settings 2. Variation in average visit duration between the field and simulation studies 3. Comparison of gaze distribution between the field and simulation settings 4. Comparison of fixation durations per second between the field and simulation settings
13, [23]	1. Post-survey results 2. GSR readings and visual attention performance of cyclists as they navigate commercial vehicle loading zones		

**Table A5:** Studies’ results.

**ID, Ref.**	**Results**	**Study ID**	**Results**
1, [9]	Cyclists’ visual behavior was not affected by listening to music.	14, [24]	- Truck maneuvers had the most significant effect on cyclists’ visual attention, and dashed green bike lanes were more effective at attracting visual attention than other pavement marking designs. - Men fixated more on pavement markings than women, regardless of truck maneuver or pavement marking type.
2, [16]	There are differences in attentional requirements and information sampling strategies between cyclists and drivers.	15, [25]	- Most of the differences in motorist performance measures were not statistically significant. - The level-one signage treatment, specifically the “Turning Vehicles Yield to Bicycles” symbol sign, positively influenced driver behavior with respect to visual attention. - The protected intersection design showed some improvements in driver performance with respect to potential crash severity, as measured by vehicle speeds in near and actual collisions.
3, [17]	- Optimal cycling conditions are when cyclists keep the balance of attention distribution between the central (trajectory) and peripheral regions of the visual field; it is reduced when pedestrian density increases. - More attention was paid to intersections and crosswalks, but the non-physical separation between cyclists and pedestrians resulted in a lack of attention to presented risks.	16, [26]	- Encountering a cyclist can evoke negative arousal in drivers: an increase in peak heart rate and electrodermal activity (EDA). - Greater attention to cyclists is associated with safer driving: evidenced is based on correlations between eye movement measures and driving performance. - Attention and physiological arousal can separately influence driving behavior: no significant correlations between physiological arousal and attention to the cyclist.
4, [19]	- The analysis of eye-tracking data allowed researchers to categorize gaze behavior and fixation patterns. - The new signage strategy was experienced as positive by most participants, and it helped them navigate the route more easily. - With the increasing complexity of the traffic environment, prioritizing route guidance information diminished.	17, [29]	- Collisions occurred when the sum of the braking reaction time (BRT) and the car braking deceleration time was larger than the time to collision (TTC) when the cyclist was visible to drivers. - As the time for participants to notice cyclists crossing from the far side was longer, it led to a longer braking response time (BRT). The study observed that participants’ behavior to avoid collisions changed from swerving to braking as the notice time increased. - Participants tended to notice the cyclist more quickly when the gaze angle was small.
5, [10]	- Cyclists faced dangers in risky weather conditions. The buildup of snow prompted cyclists to deviate from their designated cycling paths, exposing them to the risk of collisions. - Cyclists’ risky actions may pose a danger to both them and other road users. - The participants perceived greater safety in areas with dedicated cycling lanes. - Cyclists’ behavior was directly influenced by the characteristics of the road surface and the design of infrastructure geometry.	18, [30]	- Pedal cycling experience did not significantly affect drivers’ visual attention towards pedal cycles. - Drivers, in general, did not pay as much attention to pedal cycles approaching from far distances, despite them approaching at identical speeds to cars and motorcycles.
6, [11]	- The current spatial arrangement does not dictate the duration of individuals’ fixation on a specific spot. - Cyclists are required to increase their visual scanning in areas characterized by a higher number of exposed edges. - When confronted with numerous angles obscuring potential hazards, participants experienced a high visual workload, leading them to primarily focus on what lay ahead of them. - The perception of subjective risk, influenced by the spatial complexity of the location, leads to more hurried and disorganized visual scanning behavior. This includes shorter periods of fixating on objects situated closer to the individual and in directions that deviate more from the direction of travel. - Experienced cyclists exhibit a more regulated visual scanning process and demonstrate more forward-looking behavior, as indicated by longer fixation durations. Additionally, they assessed the overall test locations to be less hazardous.	19, [27]	- The presence of cyclists significantly affected driver performance, including visual attention and crash avoidance behavior. - Secondary task engagement had a significant effect on driver performance.
7, [18]	- Drivers had a high prevalence of visual scanning failures towards areas where VRUs could approach at urban intersections, particularly during right turns. - Cycling experience was found to have a moderating effect on these scanning failures, with non-cyclist drivers exhibiting more failures than cyclist drivers.	20, [28]	- The relative position of cyclists had a significant impact on drivers’ visual attention and crash risk in right-hook crash scenarios. - Drivers were more likely to detect and fixate on cyclists when they were riding ahead of the driver in their focal vision than when they were approaching from behind in the driver’s blind spot. - The presence of oncoming left-turning vehicular traffic and conflicting pedestrians in the crosswalk also increased the risk of right-hook crashes involving cyclists.
8, [12]	- Pedestrians considered relevant were the primary focus of attention regardless of the traffic signal’s status. - During red light turns, driver attention was predominantly directed towards traffic coming from the left.	21, [22]	- Physiological data, particularly changes in heart rate and gaze behavior, are sensitive to road environment changes and real-time events in the immersive virtual environment (IVE). - Different environmental factors can impact participants’ stress levels and cognitive load.
9, [13]	- There are particular locations within urban areas where cyclists are more likely to look or direct their gaze. - Cyclists present a T-shaped gaze pattern with two spots of frequent eye fixation points, which may benefit cyclists with greater safety and better readiness for road situations.	22, [21]	- The virtual reality cycling simulator provided a realistic and engaging tool to assess hazard anticipation skills in child cyclists. - Participants demonstrated delayed responses in covert hazardous situations compared to overt ones, indicating a need for improved hazard detection in certain scenarios. - Considering factors like speed and road positioning, is important in evaluating hazard anticipation skills.
10, [14]	- The number and duration of fixations varied widely based on the intersection. - Intersection design characteristics can impact cyclists’ behavior.	23, [31]	- Cyclists behaved comparably in virtual and in situ environments, and the virtual environment demonstrated potential applicability for developing hazard perception among young cyclists. - Further work is needed to fully understand the comparability of visual search behavior in both virtual and in situ settings.
11, [15]	- There were significant differences in the visual attention patterns of pedestrians, cyclists, and electric scooter riders. - The mode of transport “pedestrians” had a different pattern of visual attention compared to the other two modes. - There are dangerous riding behaviors, particularly among electric scooter riders, with speeds deemed too high to ensure pedestrians’ safety. - A total of 261 violations of traffic rules by the participants were identified, including both minor and major infractions.	24, [32]	- There are similarities between the visual behavior of participants in the simulated and real scenarios, emphasizing the validity of the use of the bicycle simulator. - The simulator was as close as possible to the real scenario, obtaining objective results very similar to each other and providing visual sensations, vibration movement, and noise.
12, [20]	- CWS usage resulted in earlier braking reactions to all investigated SCEs and partly earlier fixation on critical interaction partners (CIPs), indicating increased situation awareness among cyclists. - Participants’ assessments regarding perceived safety further supported the safety improvements derived from CWS usage independently of age group. - CWS usage did not add to mental workload ratings.	25, [33]	- The results indicated significant differences in gaze behavior between the simulator and field study. - In the simulator, the gaze distribution was more horizontal, while in the field study, it was vertical. - More fixations and visits were observed in the field study, with shorter average visit durations. - The gaze behavior was found to be more active in the field study compared to the simulation.
13, [23]	- Cyclists exhibited high visual attention to the traffic area of interest while navigating commercial vehicle loading zones. - Factors, such as the size of the loading zone, the presence of a courier, and loading zone accessories, influenced the stress levels of cyclists. - There is a correlation between perceived risk, galvanic skin response (GSR) measurements, and visual attention in commercial vehicle loading zones.		
